# Impact of Prehabilitation Components on Oxygen Uptake of People Undergoing Major Abdominal and Cardiothoracic Surgery: A Network Meta-Analysis of Randomized Controlled Trials

**DOI:** 10.3390/jcm15010175

**Published:** 2025-12-25

**Authors:** Susana Priego-Jiménez, Pablo Priego-Jiménez, María López-González, Arturo Martinez-Rodrigo, Anais López-Requena, Celia Álvarez-Bueno

**Affiliations:** 1 Hospital Universitario de Cuenca, 16003 Cuenca, Spain; susiprie@hotmail.com; 2 Center for Socio-Health Studies (CESS), Age-ABC Research Group, University of Castilla-La Mancha, 16071 Cuenca, Spain; maria.lopezgonzalez@uclm.es (M.L.-G.); anais96lopez@gmail.com (A.L.-R.); celia.alvarezbueno@uclm.es (C.Á.-B.); 3 Age-ABC Research Group, Health Research Institute of Castilla-La Mancha (IDISCAM), 45004 Toledo, Spain; 4 Department of General and Digestive Surgery, Hospital Universitario La Paz, 28046 Madrid, Spain; 5 COMETA Research Group, Informatics Systems Department, University of Castilla-La Mancha, 16071 Cuenca, Spain; arturo.martinez@uclm.es; 6 Facultad de Ciencias de la Salud, Universidad Autónoma de Chile, Talca 7500912, Chile

**Keywords:** prehabilitation, aerobic fitness, cardiorespiratory fitness, exercise training, cardiopulmonary exercise testing, nutrition, psychological, exercise capacity, oxygen uptake, VO2

## Abstract

**Background/Objectives:** Patient preoperative cardiorespiratory physical fitness measured by maximal oxygen consumption (VO2max) is highly relevant to postoperative outcomes, with low VO2max associated with a greater symptom burden and a greater prevalence of long-term treatment-related cardiovascular disease risk factors in patients undergoing surgery. A network meta-analysis (NMA) was conducted to determine the effects of different components of prehabilitation, including exercise, nutrition, psychological intervention, and different combinations of the aforementioned interventions, on oxygen consumption in people undergoing major abdominal or cardiothoracic surgery. **Methods:** A literature search was conducted from inception to December 2025. Randomized controlled trials on the effectiveness of prehabilitation programmes on pre-surgery VO2max were included. The risk of bias was assessed via the Cochrane risk of bias (RoB 2.0) tool, and the quality of evidence was assessed via the Grading of Recommendations, Assessment, Development, and Evaluation (GRADE) tool. Pairwise meta-analyses and NMAs were conducted for direct and indirect evidence. **Results:** Fourteen studies were included in this NMA. The highest effect (ES) for VO2max scores was for the exercise group versus the control group (ES: 0.44; 95% CI: 0.11, 0.78). When exercise was categorized according to intensity, the highest effect was for high-intensity interval training (HIIT) versus the control (ES: 0.51; 95% CI: 0.04, 0.97). **Conclusions:** Exercise HIIT should be considered the most effective strategy for improving exercise capacity in patients undergoing major abdominal or cardiothoracic surgery. Given the importance of VO2 as a predictor of morbidity, mortality, and the potential occurrence of adverse events after the procedure in surgical patients, it is essential to include its measurement in future studies to estimate both the risk of procedures and the effect of prehabilitation programmes.

## 1. Introduction

Recent studies suggest that the preoperative physical status of a patient is highly relevant to postoperative outcomes, with higher levels of self-reported physical activity being associated with a better postoperative course [1], and patients who present to the operating room with low functional capacity are more likely to develop postoperative complications [2,3]. Prehabilitation includes the assessment of the patient’s physical, nutritional, and psychological status with the aim of determining their functional capacity, identifying possible deficiencies, and intervening to improve their preoperative functional reserve before treatment [4,5] and avoid postoperative complications. Its efficacy has been proven in multiple studies, showing better postoperative functional capacity [6,7,8,9], nutritional [10,11] and mental status [11,12], faster recovery [9,13,14], and a shorter length of hospital stay [15]. In addition, patients in prehabilitation programmes have been shown to have fewer postoperative complications and better quality of life (QoL) [12,16,17] and physical fitness.

The occurrence of postoperative complications can have short- and long-term consequences [18], resulting in reduced QoL and a decrease in functional capacity of up to 40% [19]. Therefore, it is essential to address and attempt to modify the risk of postoperative functional deterioration in the preoperative period by optimizing resilience to the stress of surgery [20]. The adverse effects of cancer and its treatments are well known, including physical and psychosocial impairments that impact QoL [21]. The prevalence and diversity of functional impairments in people diagnosed with cancer (i.e., colorectal and oesophagogastric) has led to the need for screening and assessment, focused on interventions that seek to identify and prevent dysfunction or restore function. Advances in prehabilitation have increased in recent years, with most research focusing on exercise, with a primary focus on preoperative management [22]. However, prehabilitation has now taken a more multidisciplinary approach with components such as exercise, nutritional and psychological intervention, and cessation of habits such as alcohol or tobacco [23].

Cardiorespiratory fitness (CRF), as measured by maximal oxygen consumption (VO2max), has been proposed as an indicator of overall cardiovascular fitness [24]. Low VO2max has been associated with increased symptom burden [25] and a greater prevalence of long-term treatment-related cardiovascular disease risk factors [26] in cancer patients and is considered a strong, independent predictor of cancer, cardiovascular, and all-cause mortality [27,28]. Recent studies have revealed a significant acute and chronic reduction in VO2max after systemic [29] anticancer treatments [28,30,31]; however, the broader effects of systemic anticancer therapies on VO2max remain poorly understood. Increasing VO2max in these people, who already have a reduced VO2max, reduces surgical risk, improving the patient’s physiological reserve when undergoing surgical stress and helping to prevent potential postoperative complications. Furthermore, the use of cardiopulmonary exercise testing (CPET) to determine VO2 is not yet widespread and commonplace as a tool for evaluating patients before surgical intervention and inclusion in prehabilitation programmes.

Therefore, the main objective of this network meta-analysis (NMA) was to determine the effects of different components of prehabilitation on oxygen uptake, as measured by VO2max, in patients undergoing major abdominal or major cardiothoracic surgery. In addition, we aimed to determine whether the intensity of the exercise programmes (moderate or high intensity) could influence the improvement in VO2max.

## 2. Materials and Methods

This systematic review and NMA was reported according to the Preferred Reporting Items for Systematic Review (PRISMA-NMA) incorporating network meta-analysis (see Supplementary Table S1) [32] and was conducted following the Cochrane Collaboration Handbook [33]. The study protocol was preregistered in PROSPERO (registration number CRD420251007790).

### 2.1. Search Strategy and Selection Criteria

Two review authors (S.P.-J. and P.P.-J.) independently searched PubMed, SCOPUS, the Cochrane Central Register of Controlled Trials, the Cochrane Database of Systematic Reviews, and the Web of Science from inception to December 2025.

The aim of this study was to identify randomized controlled trials (RCTs) on the effects of different components of prehabilitation on oxygen uptake in people undergoing major abdominal or cardiothoracic surgery. The search strategy combined the relevant terms “prehabilitation”, “perioperative program”, “multimodal prehabilitation”, “surgery”, and “surgical patients”. An expert librarian was consulted, and after testing several alternatives and considering the inclusion of free terms, the search strategy that provided the most sensitivity and specificity was chosen. The full search strategy is included in the Supplementary Material (see Table S2).

In addition, the list of references included in previously published systematic reviews and meta-analyses was reviewed for potentially relevant studies.

### 2.2. Eligibility

This NMA included studies on the effectiveness of different components of prehabilitation on oxygen consumption in patients undergoing major abdominal or thoracic surgery. The inclusion criteria were as follows: (1) type of study: RCT; (2) type of participants: people undergoing major abdominal and thoracic surgery; (3) type of intervention: any component of prehabilitation programmes with the aim of improving pre-surgical oxygen consumption; (4) type of comparison: control group participants undergoing their usual practice or without a prehabilitation programme; and (5) outcomes: change in oxygen consumption, measured with CPET. No language restrictions were applied.

The exclusion criteria were (1) studies comparing the same type of prehabilitation component; (2) studies that included other types of surgery, such as knee and hip replacement, whose osteoarticular characteristics could make the development of the test difficult and biased as well as require ending the test not for physiological reasons that limited VO2 but for orthopaedic reasons; or (3) studies lacking data in selected studies to estimate the effects of the interventions. Finally, studies that did not have an RCT design were excluded.

### 2.3. Data Extraction

Two reviewers (S.P.-J. and P.P.-J.) independently extracted data from each included article: (1) study characteristics (i.e., year of publication, country, and sample size); (2) population characteristics (i.e., mean age and type of surgery); (3) intervention characteristics (i.e., prehabilitation programme components, duration, frequency, type, and modality of training in the case of exercise); and (4) outcome measurements (maximal oxygen consumption). Discrepancies in data extraction were resolved by consensus with a third reviewer (C.A.-B).

### 2.4. Categorization of the Interventions

The interventions were categorized on the basis of the components of the prehabilitation programmes included in the studies: exercise (EX), nutrition (NUT), psychological intervention (PSYCO), and the combination of the aforementioned interventions: EX + NUT, EX + PSYCO, and EX + NUT + PSYCO (multimodal). In addition, a second NMA was conducted in which the exercise intervention was divided into two categories according to exercise intensity (EX high-intensity interval training (HIIT) and EX moderate intensity (MODE)): (i) the EX component includes a personalized exercise programme that could include warm-up, strength exercises, aerobic exercise (always included), and respiratory muscle training and may include all or most of these components; (ii) the NUT includes an individualized nutritional programme adapted to the nutritional status and type of surgery or treatment to be performed and may include nutritional supplementation; (iii) the PSYCO component includes interventions aimed at reducing anxiety and stress, as well as managing emotions related to the diagnosis and treatment to be performed to improve the condition and QoL of patients; and (iv) these interventions can be studied separately in RCTs or in combination, EX + NUT and EX + PSYCO, and multimodal interventions are the most comprehensive, including all 3 interventions combined. In addition, EX interventions were subclassified on the basis of exercise intensity level: EX HIIT refers to high-intensity interval resistance training, a training modality which alternates high-intensity training with low-intensity intervals (an example of this is 80% of the maximum workload (Wpeak) at high intensity and 50% of Wpeak at low intensity), and EX MODE refers to moderate-intensity continuous resistance training, characterized by constant load throughout the exercise at a moderate intensity of 50–60%.

### 2.5. Risk of Bias Assessment

Two investigators (S.P.-J. and C.A.-B.) independently assessed the risk of bias of the included RCTs via the Cochrane Collaboration’s risk of bias (RoB2) tool [34]. Any disagreements were resolved by consensus or discussion with a third reviewer (P.P.-J.). The RoB2 tool assesses the risk of bias according to five domains: the randomization process, deviations from the intended intervention, missing outcome data, outcome measurement, and selection of reported outcomes. The overall risk of bias was classified as “low risk of bias” for studies where all domains were rated as “low risk”, “somewhat of concern” for studies where at least one domain was rated as “somewhat of concern”, and “high risk of bias” for studies where at least one domain was rated as “high risk” or where several domains were rated as “somewhat of concern”. The risk of bias assessment was performed at the study level.

### 2.6. Rating the Grade of Recommendations of the Evidence

To assess the quality of evidence and formulate recommendations, the Grading of Recommendations, Assessment, Development, and Evaluation (GRADE) tool [35] was used. On the basis of study design, risk of bias, indirect evidence, inconsistency, publication bias, and imprecision, each result was graded as high, moderate, low, or very low evidence strength, according to the assessment tool [35].

### 2.7. Data Synthesis and Statistical Analysis

To perform this NMA, we followed the steps detailed below. First, we analysed the three assumptions for NMA. (1) Similarity was checked to verify that the studies included in the NMA were similar and comparable. Samples under the same intervention were analysed and verified to be similar in baseline characteristics that could be considered confounding variables (i.e., age, percentage of women in the sample, and baseline oxygen consumption values). (2) Consistency and transitivity were checked, and the node splitting method was used to determine inconsistency. (3) Finally, heterogeneity was analysed via the I_2_ statistic, considering heterogeneity to be unimportant (I_2_: 0% and 30%), moderate (I_2_: 30–50%), substantial (I_2_: 50–75%), or considerable (I_2_: 75–100%). The size and clinical relevance of heterogeneity were determined via the τ^2^ statistic, where a τ^2^ estimate of 0.04 was interpreted as low, 0.14 as moderate, and 0.40 as a substantial degree of clinical relevance heterogeneity.

**Figure 1:**
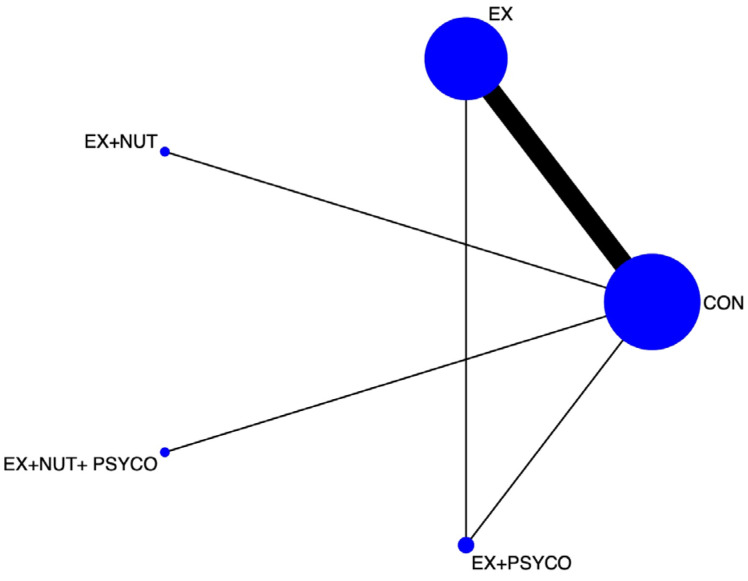
Network of available comparisons between prehabilitation components on VO2 pre-surgery. Node size is proportional to the number of trial participants, and the thickness of continuous lines connecting nodes is proportional to the number of participants randomized in trials directly comparing the two treatments EX: exercise; NUT: nutrition; PSYCO: psychological intervention.

**Figure 2:**
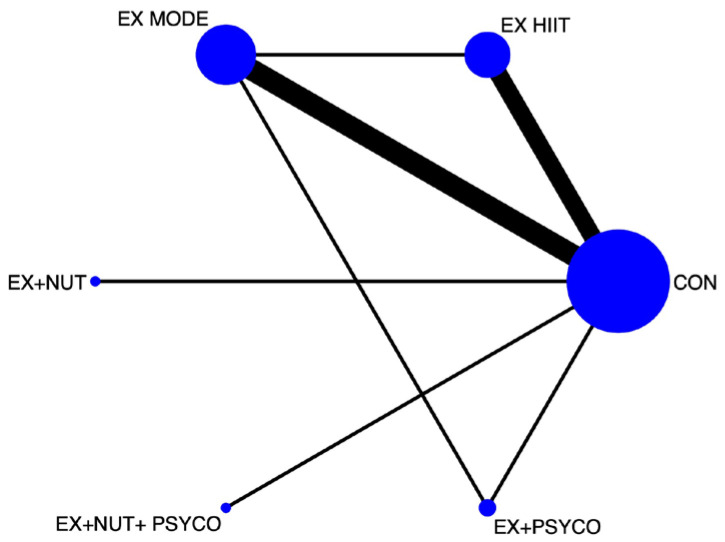
Network of available comparisons between prehabilitation components on VO2 pre-surgery, subclassifying exercise according to intensity. EX: exercise; HIIT: high-intensity interval training; MODE: moderate-intensity training.; NUT: nutrition; PSYCO: psychological intervention.

Frequentist random-effects methods were subsequently used to perform the NMA. For the study outcome (maximal oxygen consumption), the following procedures were used: (1) a network diagram to represent the included evidence (Figure 1 and Figure 2); (2) a classification table summarizing the estimates for each treatment comparison; (3) a rankogram to graphically represent the relative ranking among prehabilitation interventions; and (4) the surface under the cumulative ranking (SUCRA) to present a numerical value for each intervention’s ranking. These numbers could range from 0 to 1, with the best intervention being the closest to 1.

Finally, using DerSimonian–Laird random-effects methods, standard mean differences (ESs) were estimated for a standard pairwise meta-analysis for direct comparisons between interventions and control/no intervention. Additionally, the data for the sensitivity analyses are presented in tables, and Egger’s regression asymmetry test was used to assess publication bias. All analyses were performed via Stata 18.0.

The procedures were similar for the main NMA to analyse the effects of the different components of prehabilitation on oxygen uptake and for the subNMA to subdivide the exercise component by intensity.

Although we explored the plausibility of the transitivity assumption, differences in key effect modifiers across treatment comparisons were observed. To address this, we conducted performed subgroup analyses restricted to direct comparisons by running several subgroup meta-analyses to estimate the effects of the prehabilitation programme components based on the following variables and their classification: type of surgery (abdominal and cardiothoracic), type of pathology (oncological/non-oncological), age range of the participants included (40–59 years, 60–69 years, 70–79 years, and 80–89 years), duration of the prehabilitation intervention (<4 weeks, 4 weeks, 3–6 weeks, 6–8 weeks, and >12 weeks), type of EX intervention (END, ST, and inspiratory muscle training (IMT)), and exercise intensity in the exercise and combined interventions (MODE and HIIT), and we performed a network meta-regression for age as a continuous variable.

## 3. Results

**Figure 3:**
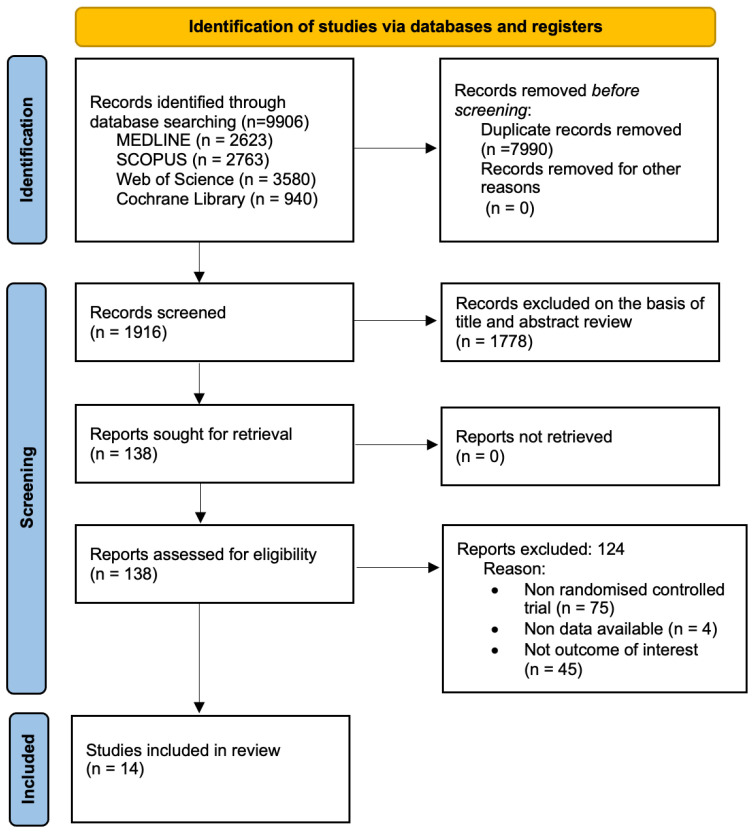
PRISMA flow diagram.

The items reported for the NMA are presented in Figure 3. Among the 9906 full-text articles identified, 14 RCTs with a total sample of 989 participants met the inclusion criteria and were included in the analyses. Among the included studies, 12 had two arms (one intervention and one control or two interventions), and 1 had three arms (two interventions and one control or one intervention and two controls). With respect to the interventions included in each study, EX + NUT was included in one study with 16 participants, EX + PSYCO was included in one study with 17 participants, and multimodal intervention (EX + NUT + PSYCO) was included in one study with 26 participants. In addition, EX HIIT was included in six studies with 230 participants, and EX MODE was included in seven studies with 226 participants. The age of the study participants ranged from 42.5 to 80 years. The characteristics of the included studies are detailed in Table 1, and the characteristics of the interventions are specified in Table 2.

### 3.1. Risk of Bias

Nine studies were assessed as having a low risk of bias, one as having some concern, and four as having a high risk of bias (Figure S1). For each specific domain, the randomization process was classified as being at low risk of bias in 78.6% of the studies; deviations from planned interventions were classified as being at low risk of bias in 92.9%; missing outcome data were classified as being at low risk of bias in 100%; outcome measurement was classified as being at low risk of bias in 78.6%; and the selection of the reported outcome was classified as being at low risk of bias in 100% (Figures S1 and S2).

Additionally, the GRADE results are available in Tables S3 and S4. The GRADE score was rated as important for all interventions except for exercise in the NMA by prehabilitation components and EX MODE in the NMA performed by intensity of exercise.

### 3.2. Network Meta-Analysis

Network diagrams showing the relative amount of evidence available on the different prehabilitation interventions for VO2 in people undergoing major abdominal or cardiothoracic surgery were constructed. The NMA by components of the prehabilitation programmes included five direct comparisons, and seven were included in the NMA after the exercise component was subdivided by intensity. Most interventions included at least one direct comparison with a control group.

Although three assumptions for NMA criteria were not met, the network was carried out, and following Cochrane recommendations, the difficulties encountered in transitivity were explained using subgroup analyses and meta-regressions (Table S5).

The highest ES for pairwise comparisons of VO2max scores was for the exercise group versus the control group (ES: 0.44; 95% CI: 0.11, 0.78) (Table 3). In the second NMA, in which exercise was categorized according to intensity, the highest ES was for EX HIIT versus the control group (ES: 0.51; 95% CI: 0.04, 0.97) (Table 4).

### 3.3. Best Treatment Probabilities

In the NMA by type of intervention, the cumulative rankogram revealed that the EX intervention had the highest probability of being classified as the first treatment estimated by the SUCRA (0.737), followed by EX + PSYCO (0.592) and EX + NUT + PSYCO (0.514). The SUCRA in the NMA by intensity of exercise showed the highest probability of being the best intervention for the EX HIIT intervention (0.737), followed by EX MODE (0.639) (Tables S8 and S9 and Figures S3 and S4).

The data from the pairwise meta-analysis are presented in Supplementary Figures S5 and S6.

The data from the pairwise meta-analysis and subgroup analyses are detailed below and presented in Supplementary Figures S7–S13 and Tables S12–S15.

Results of sensitivity analyses: In the subgroup analysis of the surgery type, the results were positive for abdominal and cardiothoracic surgery, not being statistically significant for cardiothoracic surgery and abdominal/cardiothoracic surgery (Figure S7 and Table S12). In the sub-analysis of the type of pathology, the results were positive for oncologic and non-oncologic patients, not being statistically significant for cardiothoracic surgery and abdominal/cardiothoracic surgery (Figure S8 and Table S13). For the age range of the population, the results were positive for people under 80 years of age but were only statistically significant for people under 70 years (40–59 and 60–69 years) (Figure S9 and Table S14). Meta-regression confirmed these results (Figures S10 and S11). For the duration of intervention, the results were positive for all durations of intervention, not being statistically significant for <4 weeks, 4, and 3 to 6 weeks (Figure S12 and Table S15). On the basis of the type of exercise, the best results were for END MODE + ST + PSYCO and END HIIT (Figure S13).

### 3.4. Analysis of Sensitivity, Heterogeneity, and Publication Bias

In the first NMA, EXs versus controls showed moderate heterogeneity for VO2max (I_2_ = 36.4%); when we performed a subclassification of exercise based on intensity, EX MODE versus controls showed substantial heterogeneity (I_2_ = 61.1%). The other direct comparisons did not show significant heterogeneity (P > 0.05) (Tables S16 and S17). Funnel plots are shown in Figures S14 and S15.

## 4. Discussion

The aim of this NMA was to compare the effect of each component of prehabilitation on pre-surgical oxygen consumption in patients undergoing major abdominal and cardiothoracic surgery and to determine which type of intervention is most effective in improving it. This NMA, which includes 14 RCTs and data from 989 participants, provides evidence supporting prehabilitation as an effective therapeutic strategy for improving VO2max in patients undergoing major abdominal and cardiothoracic surgery. Furthermore, the analysis of the available evidence indicates that exercise is the most effective component for improving oxygen consumption. Furthermore, when exercise by intensity (moderate and HIIT) was analysed, EX HIIT was the best intervention in improving VO2max for these patients.

Assessing exercise effort and functional reserve during exercise requires both objective and subjective tools. VO2max, considered the current gold standard for objective aerobic fitness testing, requires specialized personnel and expensive equipment, so it is not reported as regularly as other measurements, such as the 6MWT [16,40,44]. Reduced exercise capacity, as measured by oxygen uptake in older adults, limits their ability to respond to exertional stress. This limitation is associated with a corresponding limitation in their ability to respond to surgical stress, which predisposes the heart and other organ systems to injury and may contribute to older adults’ predisposition to frailty [20]. The authors associated a deterioration of VO2max with the development of adverse clinical outcomes in patients with cancer [49,50,51], which supports the recommendation of exercise to preserve and improve VO2max during and after cancer treatment.

Skeletal muscle deconditioning during systemic anticancer therapy may also contribute to CRF impairment [29]. A reduction in muscle fibre cross-sectional area has been observed, with a greater proportion of fast-twitch fibres and a decrease in mitochondrial density with altered mitochondrial function [52]. It is essential to implement strategies to prevent or reverse VO2max alterations associated with treatments and the disease process itself, with exercise training being the most effective intervention to improve VO2peak [29]. Patients included in this NMA presented fundamentally oncological and cardiothoracic pathologies, which tended to decrease patients’ CRF itself. VO2peak cut-offs of <10 mL/kg/min have been established as indicators of high surgical risk and may be an exclusion criterion from the procedure [53]; VO2max levels above 15 mL/kg/min have demonstrated better 90-day survival rates in patients undergoing cardiovascular surgery [54]. Similarly, a cut-off point of 11 in VO2 at the aerobic threshold has been associated with increased morbidity and mortality in abdominal, colorectal, biliary, urological, and vascular surgery [53].

Cancer and chronic disease patients frequently present mitochondrial dysfunction, with reduced oxidative capacity, decreased mitochondrial density, and altered energy metabolism, which contributes to decreased oxygen consumption and exercise intolerance. Exercise interventions based on strength and endurance training have emerged as effective strategies to counteract these alterations [55,56]. Physical training has been shown to regulate PGC-1α, a key regulator of mitochondrial biogenesis and oxidative metabolism [57]. Activation of PGC-1α promotes mitochondrial biogenesis and improves the efficiency of the electron transport chain and oxygen utilization by skeletal muscle, thereby increasing VO2 max [55]. Exercise in prehabilitation can partially reverse these adaptations such as cancer-related mitochondrial dysfunction and improve metabolic flexibility and cardiorespiratory capacity [58], ultimately contributing to better tolerance of surgical and oncological treatments and improved postoperative outcomes. Therefore, exercise-induced activation of PGC-1α represents a key molecular mechanism linking physical training to improved oxygen uptake and functional capacity in cancer patients undergoing prehabilitation [55,57], a phenomenon also observed in other pathologies, such as cardiorespiratory disorders [56].

There are many systematic reviews about the efficacy of prehabilitation for postoperative recovery, supporting the role of exercise as the main component of prehabilitation and reporting its efficacy in reducing postoperative stress, complications, and length of hospital stay and improving clinical outcomes by optimizing cardiopulmonary reserve before surgery [59]. However, few studies have measured VO2, probably because of both the complexity and cost of CPET, with authors establishing significant changes in VO2max between 1.14 and 4.64 mL/kg/min, which is in the range of clinically important improvements [60]. Therefore, it is necessary to assess VO2 in people undergoing major abdominal and cardiothoracic surgery, as well as to determine whether their VO2max can be improved by performing prehabilitation programmes, including exercise, and to determine which type of exercise is most effective and at what intensity to improve oxygen uptake in this specific population.

The data from this work indicate that participants involved in pre-surgical prehabilitation programmes can improve their VO2 by 1.9 mL/kg/min in people undergoing major abdominal and cardiothoracic surgery, which is in line with previous research. For many authors, VO2max is a predictor of morbidity and mortality and postoperative complications [54,61,62]. However, few studies have measured this parameter, probably because not all healthcare centres have the necessary equipment to measure this variable, as the equipment is expensive, and because of the need for trained professionals to perform the test.

### Study Limitations

This study has several limitations, in addition to those common in systematic reviews, such as language restrictions, the use of estimations instead of the original data reported in studies, and methodological issues, which should be taken into account. In addition, we were unable to consider exercise intensity in all combinations of prehabilitation components, in the network, only in cases where exercise was studied as an individual component; however, in those cases where the exercise is studied in a combined manner, we have detailed it in sub-analysis by subgroups. Second, we were unable to determine the relevance of each prehabilitation programme on the basis of disease duration; alternatively, we have performed subgroup analyses to determine the effect on the different durations of interventions included. Third, the moderate risk of bias in some of the included studies was due primarily to the difficulty of blinding the interventions; the risk of bias in the included trials was generally acceptable according to the other domains included in the RoB2 tool. Fourth, the findings should be interpreted with caution due to the limited number of studies for some interventions such as EX + NUT, EX + PSYCO, and EX + NUT + PSYCO. Further research is needed, including the assessment of VO2 in multimodal prehabilitation programmes, to allow us to refine the characteristics of the different components. Fifth, although we explored the plausibility of the transitivity assumption, differences in key effect modifiers across treatment comparisons were observed. To address this, we conducted subgroup analyses restricted to direct comparisons and performed a network meta-regression. However, as transitivity is a fundamental but untestable assumption, residual intransitivity cannot be ruled out, and the network meta-analysis results should be interpreted with caution.

## 5. Conclusions

In summary, exercise appears to be the most effective component of prehabilitation programmes for improving oxygen consumption in patients undergoing major abdominal and cardiothoracic surgery. Exercise intensity analysis concluded that EX HIIT appears to be the best intervention for improving VO2. Exercise should be considered a fundamental therapeutic strategy for patients undergoing major abdominal and cardiothoracic surgery to improve oxygen consumption. Given the importance of VO2 as a predictor of both morbidity and mortality and the potential development of adverse events after the procedure in patients undergoing surgery, it is essential to include its measurement in future studies to estimate the effect of prehabilitation programmes, as well as to further study these programmes in patients undergoing surgery. However, we must take into account the limitations in its assessment due to the still limited accessibility and difficulty in performing specific tests for the direct measurement of peak VO2, owing not only to its high cost but also to the need for professionals trained in its use.

The data from this NMA indicate the need for further research on certain interventions, such as nutritional and psychological ones, for which there is still little isolated evidence on their effectiveness as preoperative interventions, as well as the use of the CPET test to assess surgical risk, and to personalize prehabilitation interventions such as exercise, prescribed based on the results of the CPET. Based on the results obtained in our study, it appears necessary to implement and generalize prehabilitation programme protocols, standardizing the characteristics of each component and including them in the ERAS guidelines to reduce variability in outcomes, as well as incorporating oxygen consumption assessment into preoperative protocols. The results obtained can serve as a possible starting point for the implementation of these protocols.

After analysing subgroups of the different characteristics of both the interventions and the participants in the study, we can propose the ideal prehabilitation intervention for improving oxygen consumption in patients undergoing major abdominal and cardiothoracic surgery. This intervention includes at least six weeks of a combination of moderate-endurance intensity implemented by strength exercise and psychological support, or an exercise HIIT programme training, with better results with longer duration of intervention. The best results are observed in individuals under 70 years of age, particularly those undergoing abdominal surgery, regardless of whether the procedure is oncological or non-oncological.

## Figures and Tables

**Table 1:** Characteristics of the population described in the included studies. CG: control group; EX: exercise; IG: intervention group; N: sample size; NUT: nutrition; PSYCO: psychological intervention; SD: standard deviation; y: year.

Study Characteristics				Population Characteristics			
Study	Country	N	Type of Surgery	Age (y), Mean ± SD or (CI)	Groups by Intervention	VO2 Peak Baseline (mL/min)	N Intervention
Carli et al., 2010 [36]	Canada	112	Colorectal	61 ±16	**IG:** EX	1395	58
				60 ± 15	**CG:** CON	1400	54
Allen et al., 2021 [37]	UK	54	Oesophagogastric cancer	65 ±6	**IG:** EX + NUT + PSYCO	20.29 ± 4.25	26
				62 ± 9	**CG:** CON	21.28 ± 3.58	28
Bojesen et al., 2023 [38]	Denmark	36	Colorectal cancer	80 ± 6.9	**IG:** EX + NUT	12.6 ± 3.47	16
				78 ± 6.3	**CG:** CON	12.5 ± 5.47	20
Banerjee et al., 2018 [39]	UK	60	Radical cystectomy	71.60 ± 6.80	**IG:** EX	19.22 ± 4.80	30
				72.50 ± 8.40	**CG:** CON	20.38 ± 5.59	30
Dunne et al., 2016 [16]	UK	38	Colorectal liver metastasis	61 (56–66)	**IG:** EX	17.6 ± 2.3	20
				62 (53–722)	**CG:** CON	18.6 ± 3.9	17
Kim et al., 2009 [40]	Canada	21	Major bowel resection; colorectal	55 ± 15	**IG:** EX	21.5 ± 10.1	14
				65 ± 9	**CG:** CON	20.3 ± 4.6	7
Steinmetz et al., 2020 [41]	Germany	230	Coronary artery bypass graft	66.1 ± 9.0	**IG:** EX	15.7 ± 4.1	88
				67.9 ± 7.9	**CG:** CON	16.2 ± 4.1	115
Tew et al., 2012 [42]	UK	25	Small abdominal aortic aneurysm	71 ± 8	**IG:** EX	19.3 ± 4.5	11
				74 ± 6	**CG:** CON	17.9 ± 5.4	14
Barakat et al., 2016 [43]	UK	124	Elective abdominal aortic aneurysm repair	73.8 ± 6.5	**IG:** EX	18.4 ± 11.84	62
				72.9 ± 7.9	**CG:** CON	19.6 ± 11.84	62
West et al., 2014 [44]	UK	39	Rectal cancer	64 (45–82)	**IG:** EX	16.0 ± 4.3	22
				72 (62–84)	**CG:** CON	15.7 ± 5.0	17
Dronkers et al., 2010 [45]	The Netherlands	42	Abdominal oncological	71.1 ± 6.3	**IG:** EX	29.4 ± 9.5	22
				68.8 ± 6.4	**CG:** CON	31.6 ± 6.5	20
Woodfield et al., 2022 [46]	New Zealand	63	Major abdominal	66.5 ± 13.5	**IG:** EX	20.34 ± 5.211	28
				66.0 ± 15.0	**CG:** CON	21.83 ± 6.45	35
Marcon et al., 2016 [47]	Brazil	66	Bariatric	50.1 ± 2.8	**IG:** EX+ PSYCO	433.9 ± 16.8	17
				43.4 ± 2.3	**IG:** EX	435 ± 15	22
				42.5 ± 2.7	**CG:** CON	427.2 ± 16	18
Smyth et al., 2025 [48]	Irland	79	Lung and oesophagus cancer	62.07 ± 10.2	**IG:** EX	18.7 ± 5.0	42
				65.24 ± 8.2	**IG:** EX	19.6 ± 5.4	37

**Table 2:** Characteristics of the interventions described in the included studies.

Study	Groups by Intervention	Intervention	Time (min)/rep	Intensity	Duration (wk)	Frequency (x/wk)
Carli et al., 2010 [36]	**IG: EX** **CG: CON**	EX: Bike + strengthening (push-ups, sit-ups, and standing strides). Walk/breathing: Subjects were encouraged to walk daily for a minimum of 30 min + breathing ex (DB at full vital capacity and diaphragmatic breathing, huffing and coughing) + exercise to activate circulation.	Bike: 20–30 min per day. Weight training: 12 rep. 10–15 min/day. Walk: 30 min. Breath: 5 min. Ex activate circulation: 5–10 min.	Bike: 50% MHR, was increased by 10% e/w. Weight training: The weight chosen was based on people who could lift to reach volitional fatigue with 8 rep.	4	Weight training: 3 3–5 Daily
Allen et al., 2021 [37]	**IG: EX + NUT + PSYCO** **CG: CON**	EX: Warm-up + CYC + RT (6 major muscle groups using free weights and resistance bands) + home exercise programme + patient diary) + activity monitor (Fitbit Flex2^®^). NUT: Individualized dietary goals. PSYCO: Six sessions of medical coaching. Usual care, and subjects were encouraged to remain active by undertaking regular aero ex (jogging/walk/CYC) + activity monitor (Fitbit Flex2^®^).	1 h Warm-up: 5 min; CYC: 25 min; RT: 2 sets of 12 rep.	Warm-up: Very light intensity (9/20 Borg). CYC: 40–60% HRR (11–14/20 Borg) (fairly light to somewhat hard/hard). RT: 12–14 Borg (somewhat hard).	15	2 supervised + 3 home ex PSYCO: 6
Bojesen et al., 2023 [38]	**IG: EX + NUT** **CG: CON**	EX: HIIT (bike) + RT of large muscle groups. NUT: Consultation with a dietician + protein supplements + vitamins. Smoking cessation and possible drugs discontinuation or dose reduction. Standard care.	EX: HIIT: 4 HI bouts of 2–3 min, 3 min of low intensity. RT: 3 sets of 8–12 rep. NUT: 1 h consultation.	EX: HIIT: HI bouts at 90% of VO2 max low intensity at 30%.	4	EX: 3. NUT: twice/day.
Banerjee et al., 2023 [39]	**IG: EX** **CG: CON**	EX: Warm-up + CYC + cool-down. Standard care: Subjects were advised to carry on with their lifestyles in the ‘usual way’.	EX: 5–10 min warm-up + CYC (6 × 5 min intervals) + cool-down.	Warm-up (light RT: 50 W) + vigorous-intensity aero interval ex (HIIT) (13–15 Borg-70–85% predicted MHR based on 220-age) + active rest intervals (light RT: 50 W) + cool-down (low RT: 50 W).	3–6	2
Dunne et al., 2016 [16]	**IG: EX** **CG: CON**	Twelve interval ex sessions CYC (warm-up + interval training + warm-down). Standard care.	30 min IT	HIIT: Mode (<60% VO2 peak) + vigorous (>90% VO2 peak). Based on baseline CPET.	4	3
Kim et al., 2009 [40]	**IG: EX** **CG: CON**	EX: Home-based programme. Aero: Subjects were given a portable cycle ergometer + recorded training in diaries. Basic instructions to prepare for surgery.		AERO: 40–65% HRR.	4	
Steinmetz et al., 2020 [41]	**IG: EX** **CG: CON**	EX: Supervised and monitored cycle ergometer training. Two aero ex workouts with a 15 min phase of light gymnastics in between. Standard care.	EX: Two 10 min cycling workouts (1st session), gradually increased up to two 25 min cyc (6th session).	EX: 70% of VO2peak.	2	Aero: 3.
Tew et al., 2012 [42]	**IG: EX** **CG: CON**	EX: Each session involved a mixture of treadmill walking and CYC. Standard care.	EX: 35–45 min.	EX: 12–14 Borg (6–20 scale).	12	3
Barakat et al., 2016 [43]	**IG: EX** **CG: CON**	EX: Warm-up + ST + CYC + RT (heel-raise, knee extensions, dumbbells, biceps/arm curls rep, step-up lunges, knee bends) + cool-down + ST. Standard care: Continue with their normal lifestyle.	EX: 1 h. 5 min warm-up + 2 min e/e RT.	EX: Mode intensity.	6	3
West et al., 2014 [44]	**IG: EX** **CG: CON**	EX: Warm-up + cool-down + HIIT Standard care.	40 min. 5 min warm-up + 5 min cool-down.	HIIT: First sessions: 4 by 3 min interval at 80% WR at VO2 AT (mode intensity) + 4 by 2 min interval at 50% WR at VO2. between peak and AT (severe intensity). Increased to 40 min (6 × 3 intervals at mode intensity + 6 × 2 intervals at severe intensity).	6	3
Dronkers et al., 2010 [45]	**IG: EX** **CG: CON**	EX: Warm-up + RT (lower limb extensors) + IMT + aero + training functional activities + cool-down + walking or CYC for a minimum of 30 min/day + DB + diaphragmatic breathing + incentive spirometry and coughing and forced expiration techniques. Standard care: Home-based exercise advice; subjects were encouraged to be active for a minimum of 30 min/day + pedometer + DB + diaphragmatic breathing + incentive spirometry and coughing and forced expiration techniques.	60 min: 5 min warm-up + RT (1 set of 8–15 rep) + 15 min IMT + 20–30 min aero.	RT: 60–80% 1RM. IMT: 10–60% PIM. Aero: Mode intensity (55–75% MHR- 11–13 Borg).	2–4	2
Woodfield et al., 2022 [46]	**IG: EX** **CG: CON**	Warm-up + CYC + cool-down + lifestyle habits. Standard care + lifestyle habits.	30 min CYC: 5 min warm-up + 20 min + HIIT + 5 min cool-down. 5 2 min intervals followed by 1–2 min of lower intensity.	HIIT: 90% MHR + low/mode intensity. 1 min interval of HI (aim of reaching 90% MHR at least once during session) + 1 min active rest (60% MHR). 6–20 Borg.	4	
Marcon et al., 2016 [47]	**IG: EX + PSYCO** **IG: EX** **CG: CON**	EX: Aero + ST + subjects were encouraged to increase number of steps daily. PSYCO: Sessions conducted by a psychologist with strategies based on promoting and maintaining new healthy behaviours, as well as reducing undesirable behaviours (sedentary lifestyle, increased food intake, and overconsumption of carbohydrates and fat). EX: Aero + ST + subjects were encouraged to increase number of steps daily. Standard care.	EX: 25 min + 5 min ST. PSYCO: 1 h.	Borg scale: 2–4 low-to-moderate intensity.		EX: 2 PSYCO: 1 EX: 2
Smyth et al., 2025 [48]	**IG: EX** **IG: EX**	**EX HIIT:** Physiotherapist-supervised HIIT programme + standard preoperative care. **EX MODE:** Mode intensity aero ex + 3–5 ST ex targeting major muscle groups + standard preoperative care.	CYC: 5 min warm-up (50% Wpeak) + 30 min of 15 sec intervals (100% Wpeak/0 watts) + 3 min cool-down (30 watts). 20 min mode intensity.		Min 2	5 2 first weeks 3 After 2 weeks 2 in person +3 online

Abbreviatures: Aero: aerobic; AT: aerobic threshold; CPET: cardiopulmonary exercise test; CYC: cycling; DB: deep breathing; e/e: each exercise; e/w: each week; ex: exercise; HI: high-intensity; HIIT: high-intensity interval training; IMT: inspiratory muscle training; IT: interval training; MHR: maximal heart rate; min: minutes; mode: moderate; MODE: moderate intensity; PIM: maximal inspiratory pression; rep: repetitions; RT: resistance or strength training; ST: stretching; VO2: oxygen uptake; W: watts; WR: work-rate; 1RM: maximum of one repetition.

**Table 3:** Pooled mean differences of different components of prehabilitation on oxygen uptake pre-surgery.

**CONTROL**	0.25 (0.06; 0.45)	−0.01 (−0.66; 0.65)	0.09 (−0.44; 0.63)	0.75 (0.06; 1.43)
**0.44** **(0.11; 0.78)**	**EX**	NA	NA	0.60 (−0.05; 1.25)
0.06 (−1.17; 1.28)	−0.39 (−1.66; 0.88)	**EX + NUT**	NA	NA
0.24 (−0.86; 1.34)	−0.21 (−1.36; 0.94)	0.18 (−1.46; 1.83)	**EX + NUT + PSYCO**	NA
0.33 (−0.56; 1.22)	−0.11 (−1.01; 0.78)	0.27 (−1.24; 1.79)	0.09 (−1.32; 1.51)	**EX + PSYCO**

**EX:** exercise; **NA**: not applicable; **NUT:** nutrition; **PSYCO:** psychological intervention. Effect size (ES) estimates and (95% CI). Data in bold are statistically significant. The upper right triangle gives the pooled mean differences from pairwise comparisons (column intervention relative to row); the lower left triangle gives the pooled mean differences from the network meta-analysis (row intervention relative to column). The names of the interventions are in bold to better understand the table.

**Table 4:** Pooled mean differences of different components of prehabilitation on oxygen uptake pre-surgery including exercise intensity.

**CONTROL**	**0.22** **(0.02; 0.41)**	0.26 (−0.11; 0.63)	−0.01 (−0.66; 0.65)	0.09 (−0.44; 0.63)	**0.75** **(0.06; 1.43)**
**0.51** **(0.04; 0.97)**	**EX HIIT**	0.32 (−0.12; 0.77)	NA	NA	NA
0.40 (−0.04; 0.84)	−0.11 (−0.69; 0.47)	**EX MODE**	NA	NA	0.60 (−0.05; 1.25)
0.06 (−1.21; 1.33)	−0.45 (−1.80; 0.90)	−0.34 (−1.69; 1.00)	**EX + NUT**	NA	NA
0.24 (−0.91; 1.39)	−0.27 (−1.51; 0.97)	−0.16 (−1.39; 1.07)	0.18 (−1.53; 1.89)	**EX + NUT +** **PSYCO**	NA
0.31 (−0.63; 1.24)	−0.20 (−1.22; 0.83)	−0.09 (−1.03; 0.84)	0.25 (−1.33; 1.83)	0.25 (−1.33; 1.83)	**EX +** **PSYCO**

**EX:** exercise; **HIIT:** high-intensity interval training; **MODE:** moderate intensity; **NA**: not applicable; **NUT:** nutrition; **PSYCO:** psychological intervention. Effect size (ES) estimates and (95% CI). Data in bold are statistically significant. The upper right triangle gives the pooled mean differences from pairwise comparisons (column intervention relative to row); the lower left triangle gives the pooled mean differences from the network meta-analysis (row intervention relative to column). The names of the interventions are in bold to better understand the table.

## Data Availability

Data are available upon request.

## Supplementary Materials

The following supporting information can be downloaded at: https://www.mdpi.com/article/10.3390/jcm15010175/s1, Table S1: Reporting elements for systematic reviews that incorporate network meta-analysis. Table S2: Search strategy for all the databases. Table S3: Quality grading of evidence (GRADE) for VO2 pre-surgery. Table S4: Quality grading of evidence (GRADE) for VO2 pre-surgery, subclassifying exercise according to intensity. Table S5: Data for assessment of similarity assumption baseline characteristics. Table S6: Transitivity analysis data for VO2. Table S7: Transitivity analysis data for VO2 pre-surgery, subclassifying exercise according to intensity. Table S8: Surface under the cumulative ranking (SUCRA) of different components of prehabilitation on VO2 pre-surgery. Table S9: Surface under the cumulative ranking (SUCRA) of different components of prehabilitation on VO2 pre-surgery, subclassifying exercise according to intensity. Table S10: Sensitivity analysis by comparison groups (VO2 pre-surgery). Table S11: Sensitivity analysis by comparison groups (VO2 pre-surgery, subclassifying exercise according to intensity). Table S12: Subgroups analyses based on the type of surgery. Table S13: Subgroups analyses based on the type of pathology. Table S14: Pooled estimated effect size for VO2 pre-surgery depending on the age range of population. Table S15: Subgroups analyses based on the duration of intervention. Table S16: Heterogeneity statistics for each pairwise comparison (VO2 pre-surgery). Table S17: Heterogeneity statistics for each pairwise comparison (VO2 pre-surgery, subclassifying exercise according to intensity). Figure S1: Risk of bias of studies included as assessed with the RoB2 tool. Figure S2: Risk of bias assessed with RoB2 for the included studies. Figure S3: Cumulative rankogram for each component of prehabilitation on VO2 pre-surgery. EX: exercise; NUT: nutrition; PSYCO: psychological intervention. Figure S4: Cumulative rankogram for each component of prehabilitation on VO2 pre-surgery, subclassifying exercise according to intensity. EX: exercise; HIIT: high-intensity interval training; MODE: moderate-intensity training; NUT: nutrition; PSYCO: psychological intervention. Figure S5: Pooled estimated effect size for VO2 pre-surgery. Note: Positive effect size (ES) values indicate higher scores in outcomes in favour of the intervention group. EX: exercise; NUT: nutrition; PSYCO: psychological intervention. Figure S6: Pooled estimated effect size for VO2 pre-surgery, subclassifying exercise according to intensity. Note: Positive effect size (ES) values indicate higher scores in outcomes in favour of the intervention group. EX: exercise; HIIT: high-intensity interval training; MODE: moderate-intensity training; NUT: nutrition; PSYCO: psychological intervention. Figure S7: Pooled estimated effect size for VO2 pre-surgery depending on the type of surgery. Figure S8: Pooled estimated effect size for VO2 pre-surgery depending on the type of pathology oncologic or non-oncologic. Figure S9: Pooled estimated effect size for VO2 pre-surgery depending on the age range of population. Figure S10: Meta-regression for VO2 pre-surgery. Figure S11: Meta-regression for VO2 pre-surgery subclassifying exercise according to intensity. Figure S12: Pooled estimated effect size for VO2 pre-surgery depending on the duration of the intervention. Figure S13: Pooled estimated effect size for VO2 pre-surgery depending on type of exercise. Figure S14: Funnel plot for comparison-specific pooled mean differences in VO2 pre-surgery EX: exercise; NUT: nutrition; PSYCO: psychological intervention. Figure S15: Funnel plot for comparison-specific pooled mean differences in VO2 pre-surgery, subclassifying exercise according to intensity. EX: exercise; NUT: nutrition; PSYCO: psychological intervention.

## Abbreviations

The following abbreviations are used in this manuscript:

CEPT	Cardiopulmonary exercise test.
CRF	Cardiorespiratory physical fitness.
EX	Exercise.
HIIT	High-intensity interval training.
MODE	Moderate intensity.
NMA	Network meta-analysis.
NUT	Nutrition.
PA	Physical activity.
PSYCO	Psychological.
QoL	Quality of life.
RCTs	Randomized controlled trials.
VO2max	Maximal oxygen consumption.

## References

[1] Steffens D., Beckenkamp P.R., Young J., Solomon M., da Silva T.M., Hancock M.J. (2019). Is preoperative physical activity level of patients undergoing cancer surgery associated with postoperative outcomes? A systematic review and meta-analysis. Eur. J. Surg. Oncol..

[2] Lee C.H.A., Kong J.C., Ismail H., Riedel B., Heriot A. (2018). Systematic Review and Meta-analysis of Objective Assessment of Physical Fitness in Patients Undergoing Colorectal Cancer Surgery. Dis. Colon Rectum.

[3] Steffens D., Ismail H., Denehy L., Beckenkamp P.R., Solomon M., Koh C., Bartyn J., Pillinger N. (2021). Preoperative Cardiopulmonary Exercise Test Associated with Postoperative Outcomes in Patients Undergoing Cancer Surgery: A Systematic Review and Meta-Analyses. Ann. Surg. Oncol..

[4] Silver J.K., Baima J. (2013). Cancer Prehabilitation: An opportunity to decrease treatment-related morbidity, increase cancer treatment options, and improve physical and psychological health outcomes. Am. J. Phys. Med. Rehabil..

[5] Carli F., Silver J.K., Feldman L.S., McKee A., Gilman S., Gillis C., Scheede-Bergdahl C., Gamsa A., Stout N., Hirsch B. (2017). Surgical Prehabilitation in Patients with Cancer: State-of-the-science and recommendations for future research from a panel of subject matter experts. Phys. Med. Rehabil. Clin..

[6] Liu Z., Qiu T., Pei L., Zhang Y., Xu L., Cui Y., Liang N., Li S., Chen W., Huang Y. (2019). Two-Week Multimodal Prehabilitation Program Improves Perioperative Functional Capability in Patients Undergoing Thoracoscopic Lobectomy for Lung Cancer: A Randomized Controlled Trial. Anesth. Analg..

[7] Minnella E.M., Bousquet-Dion G., Awasthi R., Scheede-Bergdahl C., Carli F. (2017). Multimodal prehabilitation improves functional capacity before and after colorectal surgery for cancer: A five-year research experience. Acta Oncol..

[8] Minnella E.M., Awasthi R., Loiselle S.E., Agnihotram R.V., Ferri L.E., Carli F. (2018). Effect of Exercise and Nutrition Prehabilitation on Functional Capacity in Esophagogastric Cancer Surgery: A randomized clinical trial. JAMA Surg..

[9] Gillis C., Li C., Lee L., Awasthi R., Augustin B., Gamsa A., Liberman A.S., Stein B., Charlebois P., Feldman L.S. (2014). Prehabilitation versus rehabilitation: A randomized control trial in patients undergoing colorectal resection for cancer. Anesthesiology.

[10] Gillis C., Fenton T.R., Sajobi T.T., Minnella E.M., Awasthi R., Loiselle S., Liberman A.S., Stein B., Charlebois P., Carli F. (2019). Trimodal prehabilitation for colorectal surgery attenuates post-surgical losses in lean body mass: A pooled analysis of randomized controlled trials. Clin. Nutr..

[11] Mina D.S., Hilton W.J., Matthew A.G., Awasthi R., Bousquet-Dion G., Alibhai S.M., Au D., Fleshner N.E., Finelli A., Clarke H. (2018). Prehabilitation for radical prostatectomy: A multicentre randomized controlled trial. Surg. Oncol..

[12] Lindbäck Y., Tropp H., Enthoven P., Abbott A., Öberg B. (2018). PREPARE: Presurgery physiotherapy for patients with degenerative lumbar spine disorder: A randomized controlled trial. Spine J..

[13] Minnella E.M., Awasthi R., Bousquet-Dion G., Ferreira V., Austin B., Audi C., Tanguay S., Aprikian A., Carli F., Kassouf W. (2021). Multimodal Prehabilitation to Enhance Functional Capacity Following Radical Cystectomy: A Randomized Controlled Trial. Eur. Urol. Focus.

[14] van Rooijen S.J., Molenaar C.J., Schep G., van Lieshout R.H., Beijer S., Dubbers R., Rademakers N., Papen-Botterhuis N.E., van Kempen S., Carli F. (2019). Making Patients Fit for Surgery: Introducing a four pillar multimodal prehabilitation program in colorectal cancer. Am. J. Phys. Med. Rehabil..

[15] Gillis C., Buhler K., Bresee L., Carli F., Gramlich L., Culos-Reed N., Sajobi T.T., Fenton T.R. (2018). Effects of Nutritional Prehabilitation, With and Without Exercise, on Outcomes of Patients Who Undergo Colorectal Surgery: A Systematic Review and Meta-analysis. Gastroenterology.

[16] Dunne D.F.J., Jack S., Jones R.P., Jones L., Lythgoe D.T., Malik H.Z., Poston G.J., Palmer D.H., Fenwick S.W. (2016). Randomized clinical trial of prehabilitation before planned liver resection. Br. J. Surg..

[17] Molenaar C.J., Papen-Botterhuis N.E., Herrle F., Slooter G.D. (2019). Prehabilitation, making patients fit for surgery–a new frontier in perioperative care. Innov. Surg. Sci..

[18] Khuri S.F., Henderson W.G., DePalma R.G., Mosca C., Healey N.A., Kumbhani D.J. (2005). Determinants of Long-Term Survival After Major Surgery and the Adverse Effect of Postoperative Complications. Ann. Surg..

[19] Christensen T., Kehlet H. (1993). Postoperative fatigue. World J. Surg..

[20] Whittle J., Wischmeyer P.E., Grocott M.P., Miller T.E. (2018). Surgical Prehabilitation. Nutrition and exercise. Anesthesiol. Clin..

[21] Sitlinger A., Zafar S.Y. (2018). Health-Related Quality of Life: The impact on morbidity and mortality. Surg. Oncol. Clin..

[22] Boereboom C., Doleman B., Lund J.N., Williams J.P. (2016). Systematic review of pre-operative exercise in colorectal cancer patients. Tech. Coloproctology.

[23] Ismail H., Cormie P., Burbury K., Waterland J., Denehy L., Riedel B. (2018). Prehabilitation Prior to Major Cancer Surgery: Training for Surgery to Optimize Physiologic Reserve to Reduce Postoperative Complications. Curr. Anesthesiol. Rep..

[24] Ross R., Blair S.N., Arena R., Church T.S., Despres J.P., Franklin B.A., Haskell W.L., Kaminsky L.A., Levine B.D., Lavie C.J. (2016). Importance of Assessing Cardiorespiratory Fitness in Clinical Practice: A Case for Fitness as a Clinical Vital Sign: A Scientific Statement from the American Heart Association. Circulation.

[25] Cupit-Link M.C., Kirkland J.L., Ness K.K., Armstrong G.T., Tchkonia T., LeBrasseur N.K., Armenian S.H., Ruddy K.J., Hashmi S.K. (2017). Biology of premature ageing in survivors of cancer. ESMO Open.

[26] Jones L.W., Haykowsky M., Pituskin E.N., Jendzjowsky N.G., Tomczak C.R., Haennel R.G., Mackey J.R. (2007). Cardiovascular Reserve and Risk Profile of Postmenopausal Women After Chemoendocrine Therapy for Hormone Receptor–Positive Operable Breast Cancer. Oncologist.

[27] Groarke J.D., Payne D.L., Claggett B., Mehra M.R., Gong J., Caron J., Mahmood S.S., Hainer J., Neilan T.G., Partridge A.H. (2020). Association of post-diagnosis cardiorespiratory fitness with cause-specific mortality in cancer. Eur. Hearth J.-Qual. Care Clin. Outcomes.

[28] Jones L.W., Courneya K.S., Mackey J.R., Muss H.B., Pituskin E.N., Scott J.M., Hornsby W.E., Coan A.D., Herndon J.E., Douglas P.S. (2012). Cardiopulmonary Function and Age-Related Decline Across the Breast Cancer Survivorship Continuum. J. Clin. Oncol..

[29] Johansen S.H., Wisløff T., Edvardsen E., Kollerud S.T., Jensen J.S., Agwu G., Matsoukas K., Scott J.M., Nilsen T.S. (2025). Effects of Systemic Anticancer Treatment on Cardiorespiratory Fitness. A systematic review and meta-analysis. JACC: CardioOncology.

[30] Bellissimo M.P., Canada J.M., Jordan J.H., Ladd A.C., Heiston E.M., Brubaker P., Mihalko S.L., Reding K., D’ Agostino R., O’ Connell N. (2022). Changes in Physical Activity, Functional Capacity, and Cardiac Function during Breast Cancer Therapy. Cancer Epidemiol. Biomark. Prev..

[31] Peel A.B., Thomas S.M., Dittus K., Jones L.W., Lakoski S.G. (2014). Cardiorespiratory Fitness in Breast Cancer Patients: A Call for Normative Values. J. Am. Hearth Assoc..

[32] Hutton B., Catalá-López F., Moher D. (2016). The PRISMA statement extension for systematic reviews incorporating network meta-analysis: PRISMA-NMA. Med. Clínica.

[33] Higgins J.P.T., Thomas J., Chandler J., Cumpston M., Li T., Page M.J. (2023). Cochrane Handbook for Systematic Reviews of Interventions Version 6.4 (Updated August 2023).

[34] Sterne J.A.C., Savović J., Page M.J., Elbers R.G., Blencowe N.S., Boutron I., Cates C.J., Cheng H.Y., Corbett M.S., Eldridge S.M. (2019). RoB 2: A revised tool for assessing risk of bias in randomised trials. BMJ.

[35] Guyatt G., Oxman A.D., Akl E.A., Kunz R., Vist G., Brozek J., Norris S., Falck-Ytter Y., Glasziou P., DeBeer H. (2011). GRADE guidelines: 1. Introduction—GRADE evidence profiles and summary of findings tables. J. Clin. Epidemiol..

[36] Carli F., Charlebois P., Stein B., Feldman L., Zavorsky G., Kim D.J., Scott S., Mayo N.E. (2010). Randomized clinical trial of prehabilitation in colorectal surgery. Br. J. Surg..

[37] Allen S.K., Brown V., White D., King D., Hunt J., Wainwright J., Emery A., Hodge E., Kehinde A., Prabhu P. (2021). Multimodal Prehabilitation During Neoadjuvant Therapy Prior to Esophagogastric Cancer Resection: Effect on Cardiopulmonary Exercise Test Performance, Muscle Mass and Quality of Life—A Pilot Randomized Clinical Trial. Ann. Surg. Oncol..

[38] Bojesen R.D., Dalton S.O., Skou S.T., Jørgensen L.B., Walker L.R., Eriksen J.R., Grube C., Justesen T.F., Johansen C., Slooter G. (2023). Preoperative multimodal prehabilitation before elective colorectal cancer surgery in patients with WHO performance status I or II: Randomized clinical trial. BJS Open.

[39] Banerjee S., Manley K., Shaw B., Lewis L., Cucato G., Mills R., Rochester M., Clark A., Saxton J.M. (2017). Vigorous intensity aerobic interval exercise in bladder cancer patients prior to radical cystectomy: A feasibility randomised controlled trial. Support. Care Cancer.

[40] Kim D.J., Mayo N.E., Carli F., Montgomery D.L., Zavorsky G.S. (2009). Responsive Measures to Prehabilitation in Patients Undergoing Bowel Resection Surgery. Tohoku J. Exp. Med..

[41] Steinmetz C., Bjarnason-Wehrens B., Baumgarten H., Walther T., Mengden T., Walther C. (2020). Prehabilitation in patients awaiting elective coronary artery bypass graft surgery–effects on functional capacity and quality of life: A randomized controlled trial. Clin. Rehabil..

[42] Tew G.A., Moss J., Crank H., Mitchell P.A., Nawaz S. (2012). Endurance Exercise Training in Patients with Small Abdominal Aortic Aneurysm: A Randomized Controlled Pilot Study. Arch. Phys. Med. Rehabil..

[43] Barakat H.M., Shahin Y., Khan J.A., McCollum P.T., Chetter I.C. (2016). Preoperative Supervised Exercise Improves Outcomes After Elective Abdominal Aortic Aneurysm Repair. Ann. Surg..

[44] West M., Loughney L., Lythgoe D., Barben C., Sripadam R., Kemp G., Grocott M., Jack S. (2015). Effect of prehabilitation on objectively measured physical fitness after neoadjuvant treatment in preoperative rectal cancer patients: A blinded interventional pilot study. Br. J. Anaesth..

[45] Dronkers J.J., Lamberts H., Reutelingsperger I.M.M.D., Naber R.H., Dronkers-Landman C.M., Veldman A., Van Meeteren N.L.U. (2010). Preoperative therapeutic programme for elderly patients scheduled for elective abdominal oncological surgery: A randomized controlled pilot study. Clin. Rehabil..

[46] Woodfield J.C., Clifford K., Wilson G.A., Munro F., Baldi J.C. (2022). Short-term high-intensity interval training improves fitness before surgery: A randomized clinical trial. Scand. J. Med. Sci. Sports.

[47] Marcon E.R., Baglioni S., Bittencourt L., Lopes C.L.N., Neumann C.R., Trindade M.R.M. (2016). What Is the Best Treatment before Bariatric Surgery? Exercise, Exercise and Group Therapy, or Conventional Waiting: A Randomized Controlled Trial. Obes. Surg..

[48] Smyth E., O’Neill L.M., Kearney N., Sheill G., Brennan L., Wade S. (2025). Preoperative exercise to improve fitness in patiens undergoing complex surgery for cancer of the lung or esophagus (PRE-HII). A randomized controlled trial. Ann. Surg..

[49] Ligibel J.A., Bohlke K., May A.M., Clinton S.K., Demark-Wahnefried W., Gilchrist S.C., Irwin M.L., Late M., Mansfield S., Marshall T.F. (2022). Exercise, Diet, and Weight Management During Cancer Treatment: ASCO Guideline. J. Clin. Oncol..

[50] Campbell K.L., Winters-Stone K.M., Wiskemann J., May A.M., Schwartz A.L., Courneya K.S., Zucker D.S., Matthews C.E., Ligibel J.A., Gerber L.H. (2019). Exercise Guidelines for Cancer Survivors: Consensus Statement from International Multidisciplinary Roundtable. Med. Sci. Sports Exerc..

[51] Gilchrist S.C., Barac A., Ades P.A., Alfano C.M., Franklin B.A., Jones L.W., La Gerche A., Ligibel J.A., Lopez G., Madan K. (2019). Cardio-Oncology Rehabilitation to Manage Cardiovascular Outcomes in Cancer Patients and Survivors: A Scientific Statement from the American Heart Association. Circulation.

[52] Mijwel S., Cardinale D.A., Norrbom J., Chapman M., Ivarsson N., Wengström Y., Sundberg C.J., Rundqvist H. (2018). Exercise training during chemotherapy preserves skeletal muscle fiber area, capillarization, and mitochondrial content in patients with breast cancer. FASEB J..

[53] Zagolin M., Trujillo L.M., Villanueva S., Ruiz M., VON Oetinger A. (2020). Test cardiopulmonar: Una herramienta de utilidad diagnóstica y pronóstica. Rev. Medica De Chile.

[54] Moran J., Wilson F., Guinan E., McCormick P., Hussey J., Moriarty J. (2016). The preoperative use of field tests of exercise tolerance to predict postoperative outcome in intra-abdominal surgery: A systematic review. J. Clin. Anesth..

[55] Hood D.A., Uguccioni G., Vainshtein A., D’souza D. (2011). Mechanisms of exercise-induced mitochondrial biogenesis in skeletal muscle: Implications for health and disease. Compr Physiol..

[56] Priego-Jiménez S., Lucerón-Lucas-Torres M., Ruiz-Grao M.C., Guzmán-Pavón M.J., Lorenzo-García P., Araya-Quintanilla F., Álvarez-Bueno C. (2024). Effect of exercise interventions on oxygen uptake in people with chronic obstructive pulmonary disease: A network meta-analysis of randomized controlled trials. Ann. Phys. Rehabil. Med..

[57] Handschin C., Spiegelman B.M. (2008). The role of exercise and PGC1α in inflammation and chronic disease. Nature.

[58] San-Millán I. (2023). The Key Role of Mitochondrial Function in Health and Disease. Antioxidants.

[59] Carli F., Scheede-Bergdahl C. (2015). Prehabilitation to Enhance Perioperative Care. Anesthesiol. Clin..

[60] Jones L.W., Liang Y., Pituskin E.N., Battaglini C.L., Scott J.M., Hornsby W.E., Haykowsky M. (2011). Effect of Exercise Training on Peak Oxygen Consumption in Patients with Cancer: A Meta-Analysis. Oncologist.

[61] Snowden C.P., Prentis J.F., Jacques B.F., Anderson H.F., Manas D.F., Jones D., Trenell M. (2013). Cardiorespiratory Fitness Predicts Mortality and Hospital Length of Stay After Major Elective Surgery in Older People. Ann. Surg..

[62] Rodríguez-Arias F.L., Sánchez-Guillén L., Ruiz L.I.A., Lara C.D., Gómez F.J.L., Pons C.B., Rodríguez J.M.R., Arroyo A. (2020). Revisión narrativa de la prehabilitación en cirugía: Situación actual y perspectivas futuras. Cir. Esp..

