# Supplementary material for: Impact of Prehabilitation Components on Oxygen Uptake of People Undergoing Major Abdominal and Cardiothoracic Surgery: A Network Meta-Analysis of Randomized Controlled Trials
Source: J Clin Med. 2025 Dec 25;15(1):175. doi: 10.3390/jcm15010175 (PMC12786905; doi:10.3390/jcm15010175)
Supplement: Supplementary file 1 [file jcm-15-00175-s001.zip › jcm-4017143-supplementary.pdf]

## SUPPLEMENTARY MATERIAL

**Table S.1. PRISMA checklist.** Reporting Elements for Systematic Reviews that incorporate Network Meta-Analysis**.**

| Section/Topic | Item # | Checklist Item | Reported on Page # |
|---|---|---|---|
| **TITLE** | | | |
| Title | 1 | Identify the report as a systematic review *incorporating a network meta-analysis (or related form of meta-analysis).* | ***1*** |
| **ABSTRACT** | | | |
| Structured summary | 2 | Provide a structured summary including as applicable:<br>**Background:** main objectives<br>**Methods:** data sources; study eligibility criteria, participants, and interventions; study appraisal; and *synthesis methods, such as network meta-analysis.*<br>**Results:** number of studies and participants identified; summary estimates with corresponding confidence/credible intervals; *treatment rankings may also be discussed. Authors may choose to summarize pairwise comparisons against a chosen treatment included in their analyses for brevity.*<br>**Discussion/Conclusions:** limitations; conclusions and implications of findings.<br>**Other:** primary source of funding; systematic review registration number with registry name. | ***1*** |
| **INTRODUCTION** | | | |
| Rationale | 3 | Describe the rationale for the review in the context of what is already known*, including mention of why a network meta-analysis has been conducted.* | ***2*** |
| Objectives | 4 | Provide an explicit statement of questions being addressed, with reference to participants, interventions, comparisons, outcomes, and study design (PICOS). | ***2,3*** |
| **METHODS** | | | |
| Protocol and registration | 5 | Indicate whether a review protocol exists and if and where it can be accessed (e.g., Web address); and, if available, provide registration information, including registration number. | ***3*** |
| Eligibility criteria | 6 | Specify study characteristics (e.g., PICOS, length of follow-up) and report characteristics (e.g., years considered, language, publication status) used as criteria for eligibility, giving rationale. *Clearly, describe eligible treatments included in the treatment network and note whether any have been clustered or merged into the same node (with justification).* | ***3*** |
| Information sources | 7 | Describe all information sources (e.g., databases with dates of coverage, contact with study authors to identify additional studies) in the search and date last searched. | ***3*** |
| Search | 8 | Present full electronic search strategy for at least one database, including any limits used, such that it could be repeated. | ***3 and table S2*** |
| Study selection | 9 | State the process for selecting studies (i.e., screening, eligibility, included in systematic review, and, if applicable, included in the meta-analysis). | ***3*** |
| Data collection process | 10 | Describe method of data extraction from reports (e.g., piloted forms, independently, in duplicate) and any processes for obtaining and confirming data from investigators. | ***3,4*** |
| Data items | 11 | List and define all variables for which data were sought (e.g., PICOS, funding sources) and any assumptions and simplifications made. | ***4*** |

| | | | |
|---|---|---|---|
| **Geometry of the network** | **S1** | Describe methods used to explore the geometry of the treatment network under study and potential biases related to it. This should include how the evidence base has been graphically summarized for presentation, and what characteristics were compiled and used to describe the evidence base to readers. | ***5,6*** |
| Risk of bias within individual studies | 12 | Describe methods used for assessing risk of bias of individual studies (including specification of whether this was done at the study or outcome level), and how this information is to be used in any data synthesis. | ***5,7*** |
| Summary measures | 13 | State the principal summary measures (e.g., risk ratio, difference in means). *Also describe the use of additional summary measures assessed, such as treatment rankings and surface under the cumulative ranking curve (SUCRA) values, as well as modified approaches used to present summary findings from meta-analyses.* | ***5,7*** |
| Planned methods of analysis | 14 | Describe the methods of handling data and combining results of studies for each network meta-analysis. This should include, but not be limited to:<br>• *Handling of multiarm trials;*<br>• *Selection of variance structure;*<br>• *Selection of prior distributions in Bayesian analyses; and*<br>• *Assessment of model fit.* | ***5,6*** |
| **Assessment of Inconsistency** | **S2** | Describe the statistical methods used to evaluate the agreement of direct and indirect evidence in the treatment network(s) studied. Describe efforts taken to address its presence when found. | ***5-7*** |
| Risk of bias across studies | 15 | Specify any assessment of risk of bias that may affect the cumulative evidence (e.g., publication bias, selective reporting within studies). | ***4,6*** |
| Additional analyses | 16 | Describe methods of additional analyses if done, indicating which were prespecified. This may include, but not be limited to, the following:<br>• Sensitivity or subgroup analyses;<br>• Meta-regression analyses;<br>• *Alternative formulations of the treatment network; and*<br>• *Use of alternative prior distributions for Bayesian analyses (if applicable).* | ***6,7*** |
| **RESULTS†** | | | |
| Study selection | 17 | Give numbers of studies screened, assessed for eligibility, and included in the review, with reasons for exclusions at each stage, ideally with a flow diagram. | ***6 and Figure 1*** |
| **Presentation of network structure** | **S3** | Provide a network graph of the included studies to enable visualization of the geometry of the treatment network. | ***Figures 2,3*** |
| **Summary of network geometry** | **S4** | Provide a brief overview of characteristics of the treatment network. This may include commentary on the abundance of trials and randomized patients for the different interventions and pairwise comparisons in the network, gaps of evidence in the treatment network, and potential biases reflected by the network structure. | ***5,6*** |
| Study characteristics | 18 | For each study, present characteristics for which data were extracted (e.g., study size, PICOS, follow-up period) and provide the citations. | ***Tables 1 and 2*** |
| Risk of bias within studies | 19 | Present data on risk of bias of each study and, if available, any outcome level assessment. | ***4,6*** |
| Results of individual studies | 20 | For all outcomes considered (benefits or harms), present, for each study: 1) simple summary data for each intervention group, and 2) effect estimates and confidence intervals. *Modified approaches may be needed to deal with information from larger networks.* | ***6,7 and figure 1 and 2 supplementary material*** |

| | | | |
|---|---|---|---|
| Synthesis of results | 21 | Present results of each meta-analysis done, including confidence/credible intervals. *In larger networks, authors may focus on comparisons* versus *a particular comparator (e.g. placebo or standard care), with full findings presented in an appendix. League tables and forest plots may be considered to summarize pairwise comparisons.* If additional summary measures were explored (such as treatment rankings), these should also be presented. | ***Table 3,4*** |
| **Exploration for inconsistency** | **S5** | Describe results from investigations of inconsistency. This may include such information as measures of model fit to compare consistency and inconsistency models, *P* values from statistical tests, or summary of inconsistency estimates from different parts of the treatment network. | ***7, Supplementary Table 6 and 7*** |
| Risk of bias across studies | 22 | Present results of any assessment of risk of bias across studies for the evidence base being studied. | ***6*** |
| Results of additional analyses | 23 | Give results of additional analyses, if done (e.g., sensitivity or subgroup analyses, meta-regression analyses, *alternative network geometries studied, alternative choice of prior distributions for Bayesian analyses,* and so forth). | ***7*** |
| **DISCUSSION** | | | |
| Summary of evidence | 24 | Summarize the main findings, including the strength of evidence for each main outcome; consider their relevance to key groups (e.g., healthcare providers, users, and policy-makers). | ***7,8,9*** |
| Limitations | 25 | Discuss limitations at study and outcome level (e.g., risk of bias), and at review level (e.g., incomplete retrieval of identified research, reporting bias). *Comment on the validity of the assumptions, such as transitivity and consistency. Comment on any concerns regarding network geometry (e.g., avoidance of certain comparisons).* | ***9*** |
| Conclusions | 26 | Provide a general interpretation of the results in the context of other evidence, and implications for future research. | ***9*** |
| **FUNDING** | | | |
| Funding | 27 | Describe sources of funding for the systematic review and other support (e.g., supply of data); role of funders for the systematic review. This should also include information regarding whether funding has been received from manufacturers of treatments in the network and/or whether some of the authors are content experts with professional conflicts of interest that could affect use of treatments in the network. | ***No funding*** |

PICOS = population, intervention, comparators, outcomes, study design.

* Text in italics indicates wording specific to reporting of network meta-analyses that has been added to guidance from the PRISMA statement.

† Authors may wish to plan for use of appendices to present all relevant information in full detail for items in this section.

**Table S.2**. Search strategy for all the databases.

| ("prehabilitation" OR "perioperative program" OR “multimodal prehabilitation”) and ("surgery" OR “surgical patients” OR “cancer”) |
|---|

“A total of 9906 records were retrieved from four databases (MEDLINE, SCOPUS, Web of Science and the Cochrane Library). After removing 7990 duplicates via automation tools (Mendeley), unique titles/abstracts 1916 were screened; 1778 were excluded on the basis of title and abstract review by human searchers. A total of 138 full-text articles were assessed, and 124 were excluded because they were nonrandomized controlled trial designs (n=75) or did not include the available data (n=4) or variables of interest (n=45). Fourteen RCTs met the inclusion criteria and were included in the quantitative synthesis.”

| Studies with pre-protocol | Unique ID | Outcome Weight | | Randomization process | Deviations from intended interventions | Missing outcome data | Measurement of the outcome | Selection of reporteed studes | Overall Bias |
|---|---|---|---|---|---|---|---|---|---|
| Carli et al; 2010 | | 1 | + | + | + | Ñ | + | Ñ | |
| Allen et al; 2021 | | 1 | + | ? | + | + | + | ! | |
| Bojesen et al; 2023 | | 1 | + | + | + | + | + | + | |
| Banerjee et al; 2018 | | 1 | + | + | + | + | + | + | |
| Dunne et al; 2016 | | 1 | + | + | + | + | + | + | |
| Kim et al; 2009 | | 1 | ? | + | + | Ñ | + | Ñ | |
| Tew et al; 2012 | | 1 | + | + | + | + | + | + | |
| Steinmetz et al; 2020 | | 1 | ? | + | + | Ñ | + | Ñ | |
| Barakat et al; 2016 | | 1 | + | + | + | + | + | + | |
| West et al; 2014 | | 1 | Ñ | + | + | + | + | Ñ | |
| Dronkers et al; 2010 | | 1 | + | + | + | + | + | + | |
| Marcon et al; 2016 | | 1 | + | + | + | + | + | + | |
| Woodfield et al; 2022 | | 1 | + | + | + | + | + | + | |
| Smyth et al; 2025 | | 1 | + | + | + | + | + | + | |

\+ Low risk
? Some concerns
Ñ High risk

**Figure S.1.** Risk of bias of studies included as assessed with the RoB2 tool.

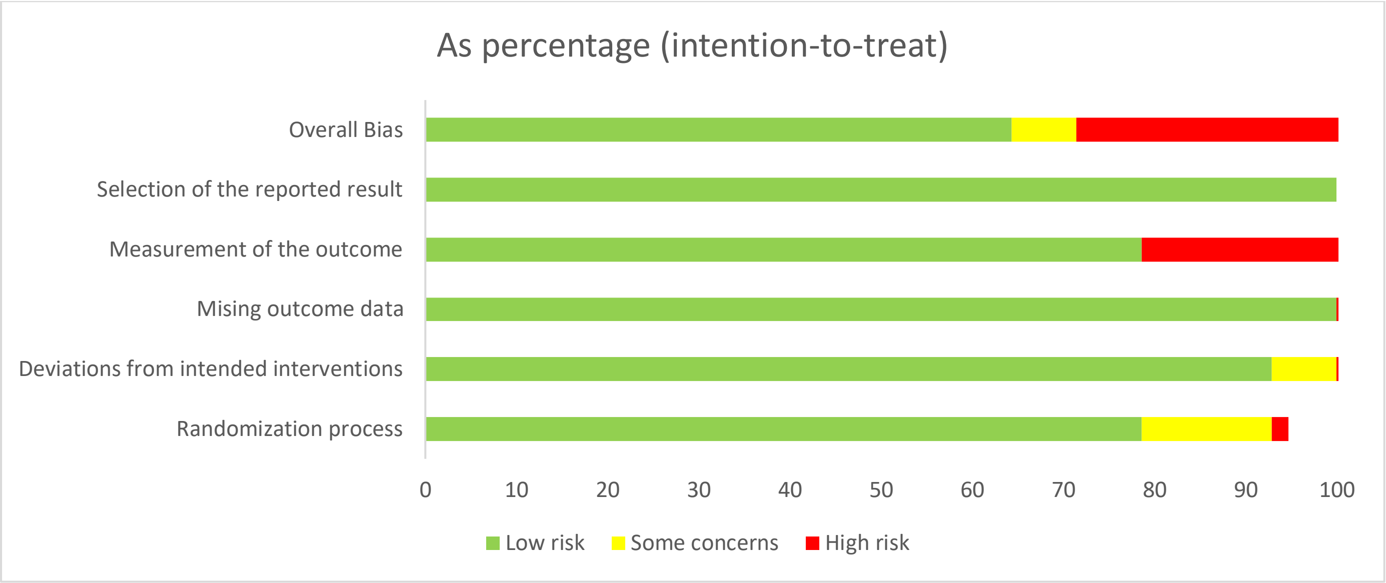


**Figure S.2.** Risk of bias assessed with RoB2 for the included studies.

**Table S.3.** Quality grading of evidence (GRADE) for VO2 presurgery

| Certainty assessment | | | | | | № of participants | | Effect | |
|---|---|---|---|---|---|---|---|---|---|
| **№ of studies** | **Study design** | **Risk of bias** | **Inconsistency** | **Indirectness** | **Imprecision** | **Intervention** | **Control** | **Relative (95% CI)** | **Importance** |
| **EX versus CONTROL** | | | | | | | | | |
| 11 | randomized trials | not serious | not serious | not serious | not serious | 377/766 (49.2%) | 389/766 (50.8%) | **0.40** (0.11; 0.62) | CRITICAL |
| **EX+NUT+PSYCO versus CONTROL** | | | | | | | | | |
| 1 | randomized trials | serious[a] | not serious | not serious | not serious | 26/54 (48.1%) | 28/54 (51.9%) | **0.09** (-0.44; 0.63) | IMPORTANT |
| **EX+NUT versus CONTROL** | | | | | | | | | |
| 1 | randomized trials | serious[b] | serious[c] | not serious | not serious | 16/36 (44.4%) | 20/36 (55.6%) | **-0.01** (-0.66; 0.65) | IMPORTANT |
| **EX versus EX+PSYCO** | | | | | | | | | |
| 1 | randomized trials | serious[b] | not serious | not serious | not serious | 22/39 (56.4%) | 17/39 (43.6%) | **0.60** (-0.05; 1.25) | IMPORTANT |
| **EX+PSYCO versus CONTROL** | | | | | | | | | |
| 1 | randomized trials | serious[b] | serious[c] | not serious | not serious | 17/35 (48.6%) | 18/35 (51.4%) | **0.75** (0.06; 1.43) | IMPORTANT |

**CI:** confidence interval.

**Explanations:**

a. The articles included present some concerns of risk; b. We have only found one article comparing these two interventions; c. the results obtained in the meta-analysis and in the network do not go in the same direction.

**Table S.4.** Quality grading of evidence (GRADE) for VO2 presurgery, subclassifying exercise according to intensity.

| Certainty assessment | | | | | | № of participants | | Effect | |
|---|---|---|---|---|---|---|---|---|---|
| **№ of studies** | **Study design** | **Risk of bias** | **Inconsistency** | **Indirectness** | **Imprecision** | **Intervention** | **Control** | **Relative (95% CI)** | **Importance** |
| **EX MODE versus CONTROL** | | | | | | | | | |
| 6 | randomized trials | not serious | not serious | not serious | not serious | 189/364 (51.9%) | 175/364 (48.1%) | **0.26** (-0.11; 0.63) | CRITICAL |
| **EX+NUT+PSYCO versus CONTROL** | | | | | | | | | |
| 1 | randomized trials | serious[a] | not serious | not serious | not serious | 26/54 (48.1%) | 28/54 (51.9%) | **0.09** (-0.44; 0.63) | IMPORTANT |
| **EX+NUT versus CONTROL** | | | | | | | | | |
| 1 | randomized trials | serious[b] | serious[c] | not serious | not serious | 16/36 (44.4%) | 20/36 (55.6%) | **-0.01** (-0.66; 0.65) | IMPORTANT |
| **EX HIIT versus CONTROL** | | | | | | | | | |
| 5 | randomized trials | not serious | serious[c] | not serious | not serious | 188/402 (46.8%) | 214/402 (53.2%) | **0.22** (0.02; 0.41) | IMPORTANT |
| **EX MODE versus EX+PSYCO** | | | | | | | | | |
| 1 | randomized trials | serious[b] | serious[c] | not serious | not serious | 22/29 (75.9%) | 17/39 (43.6%) | **0.60** (-0.05; 1.25) | IMPORTANT |
| **EX+PSYCO versus CONTROL** | | | | | | | | | |
| 1 | randomized trials | serious[b] | serious[c] | not serious | not serious | 17/35 (48.6%) | 18/35 (51.4%) | **0.75** (0.06; 1.43) | IMPORTANT |
| **EX HIIT versus EX MODE** | | | | | | | | | |
| 1 | randomized trials | serious[b] | serious[c] | not serious | not serious | 42/79 (53.2%) | 37/79 (46.8%) | **0.32** (-0.12; 0.77) | IMPORTANT |

**CI:** confidence interval; **HIIT:** high-intensity interval training; **MODE:** moderate intensity.

**Explanations:**

a. The articles included present some concerns of risk; b. We have only found one article comparing these two interventions; c. the results obtained in the meta-analysis and in the network do not go in the same direction.

**Table S.5.** Data for assessment of similarity assumption baseline characteristics

| Intervention | | Age | VO2 (ml/kg/min) (ml/min) | %women |
|---|---|---|---|---|
| Control | N | 13 | 13 | 7 |
| | Mean | 66,31 | 125,44 | 34,29 |
| | SD | 9,05 | 382,99 | 21,01 |
| Exercise HIIT | N | 6 | 6 | 4 |
| | Mean | 65,00 | 17,93 | 27,50 |
| | SD | 3,58 | 1,84 | 12,07 |
| Exercise MODE | N | 7 | 7 | 1 |
| | Mean | 62,86 | 216,87 | 36,00 |
| | SD | 10,96 | 519,52 | - |
| Exercise+ Nutrition | N | 1 | 1 | 1 |
| | Mean | 80,00 | 12,60 | 31,00 |
| | SD | - | - | - |
| Exercise + Nutrition+ Psychological | N | 1 | 1 | 1 |
| | Mean | 65,00 | 20,29 | 15,00 |
| | SD | - | - | - |
| Exercise + Psychological | N | 1 | 1 | 1 |
| | Mean | 50,00 | 14,90 | 100 |
| | SD | - | - | - |

**HIIT:** high-intensity interval training; **MODE:** moderate intensity.

**Table S.6.** Transitivity analysis data for VO2.

| Loop | IF | SE IF | z_value | p | 95%CI | $\tau^2$ |
|---|---|---|---|---|---|---|
| CONTROL-EXERCISE- EXERCISE+PSYCHOLOGICAL | 1.175 | 0.487 | 2.414 | 0.016 | (0.22; 2.13) | 0.000 |

**Table S.7.** Transitivity analysis data for VO2 presurgery, subclassifying exercise according to intensity.

| Loop | IF | SE IF | z_value | p | 95%CI | $\tau^2$ |
|---|---|---|---|---|---|---|
| CONTROL-EXERCISE MODE- EX+PSYCHOLOGICAL | 1.228 | 0.494 | 2.486 | 0.013 | (0.26; 2.20) | 0.000 |
| CONTROL-EXERCISE HIIT- EX MODE | 0.303 | 0.413 | 0.733 | 0.464 | (0.00; 1.11) | 0.051 |

**Table S.8.** Surface under the cumulative ranking (SUCRA) of different components of prehabilitation on VO2 presurgery.

| Intervention | Rank statistics | | | Probabilities | |
|---|---|---|---|---|---|
| | Mean | Median | ES | Best | SUCRA |
| CONTROL | 4.0 | 2.00 | 0.84 | 0.1 | 0.259 |
| EXERCISE | 2.1 | 1.00 | 0.98 | 28.6 | 0.737 |
| EXERCISE+NUTRITION | 3.4 | 4.00 | 0.68 | 18.7 | 0.399 |
| EXERCISE+NUTRITION+PSYCHOLOGICAL | 2.9 | 3.00 | 0.32 | 25.7 | 0.514 |
| EXERCISE +PSYCHOLOGICAL | 2.6 | 3.00 | 0.37 | 26.9 | 0.592 |

**ES:** Effect size

**Table S.9.** Surface under the cumulative ranking (SUCRA) of different components of prehabilitation on VO2 presurgery, subclassifying exercise according to intensity.

| Intervention | Rank statistics | | | Probabilities | |
|---|---|---|---|---|---|
| | Mean | Median | ES | Best | SUCRA |
| CONTROL | 3.7 | 4.00 | 1.05 | 0.0 | 0.224 |
| EXERCISE HIIT | 3.5 | 3.50 | 0.99 | 28.5 | 0.737 |
| EXERCISE MODE | 3.3 | 3.50 | 0.76 | 12.5 | 0.639 |
| EXERCISE+NUTRITION | 4.0 | 4.50 | 0.73 | 15.6 | 0.375 |
| EXERCISE+NUTRITION+PSYCHOLOGICAL | 3.2 | 3.50 | 0.40 | 22.5 | 0.494 |
| EXERCISE +PSYCHOLOGICAL | 3.3 | 3.00 | 0.21 | 20.9 | 0.532 |

**ES:** effect size; **HIIT:** high-intensity interval training; **MODE:** moderate-intensity training.

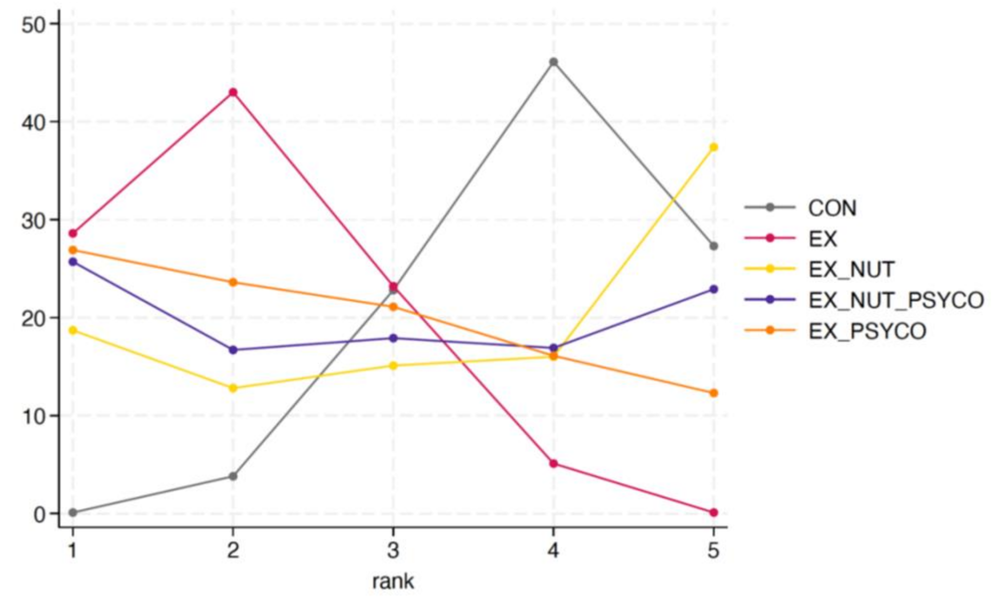


**Figure S.3** Cumulative rankogram for each component of prehabilitation on VO2 presurgery. **EX:** exercise; **NUT**: nutrition; **PSYCO:** psychological intervention.

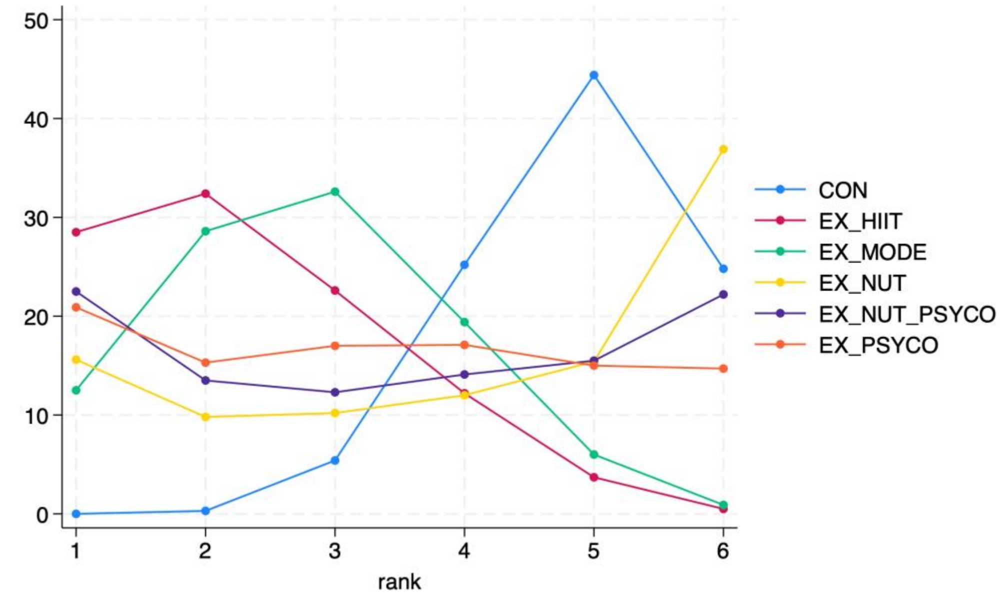


**Figure S.4** Cumulative rankogram for each component of prehabilitation on VO2 presurgery, subclassifying exercise according to intensity. **EX:** exercise; **HIIT:** high-intensity interval training; **MODE:** moderate-intensity training; **NUT**: nutrition; **PSYCO:** psychological intervention**.**

**Table S.10.** Sensitivity analysis by comparison groups (VO2 presurgery)

***Exercise group* vs *control group***

| Reference | ES | LL | UL |
|---|---|---|---|
| Carli et al., 2010 | 0.255 | 0.061 | 0.448 |
| Banerjee et al; 2018 | 0.267 | 0.052 | 0.483 |
| Dunne et al; 2016 | 0.273 | 0.047 | 0.498 |
| Kim et al; 2009 | 0.269 | 0.045 | 0.494 |
| Steinmetz et al., 2020 | 0.290 | 0.101 | 0.479 |
| Tew et al., 2012 | 0.249 | 0.040 | 0.458 |
| Barakat et al; 2016 | 0.269 | 0.065 | 0.473 |
| West et al; 2014 | 0.173 | 0.026 | 0.320 |
| Dronkers et al; 2010 | 0.293 | 0.069 | 0.516 |
| Woodfield et al; 2022 | 0.261 | 0.053 | 0.468 |
| Marcon et al; 2016 | 0.227 | 0.028 | 0.426 |

**ES:** effect size; **LL:** lower limit; **UL:** upper limit.

**Table S.11.** Sensitivity analysis by comparison groups (VO2 presurgery, subclassifying exercise according to intensity).

***Exercise MODE group* vs *control group***

| Reference | ES | LL | UL |
|---|---|---|---|
| Carli et al., 2010 | 0.259 | -0.110 | 0.628 |
| Kim et al; 2009 | 0.284 | -0.225 | 0.794 |
| Tew et al., 2012 | 0.280 | -0.226 | 0.785 |
| Barakat et al., 2016 | 0.364 | -0.023 | 0.751 |
| Dronkers et al; 2010 | 0.295 | -0.118 | 0.708 |
| Marcon et al; 2016 | 0.120 | -0.099 | 0.339 |

**ES:** Effect size; **LL:** Low limit; **MODE:** moderate-intensity training.**;** **UL:** Upper limit

***Exercise HIIT* vs *control group***

| Reference | ES | LL | UL |
|---|---|---|---|
| Banerjee et al., 2018 | 0.216 | 0.018 | 0.413 |
| Dunne et al; 2016 | 0.221 | 0.007 | 0.436 |
| Steinmetz et al., 2020 | 0.199 | -0.008 | 0.407 |
| West et al., 2014 | 0.350 | 0.068 | 0.631 |
| Woodfield et al; 2022 | 0.173 | -0.034 | 0.381 |

**ES:** Effect size; **HIIT:** high-intensity interval training; **LL:** Low limit; **UL:** Upper limit

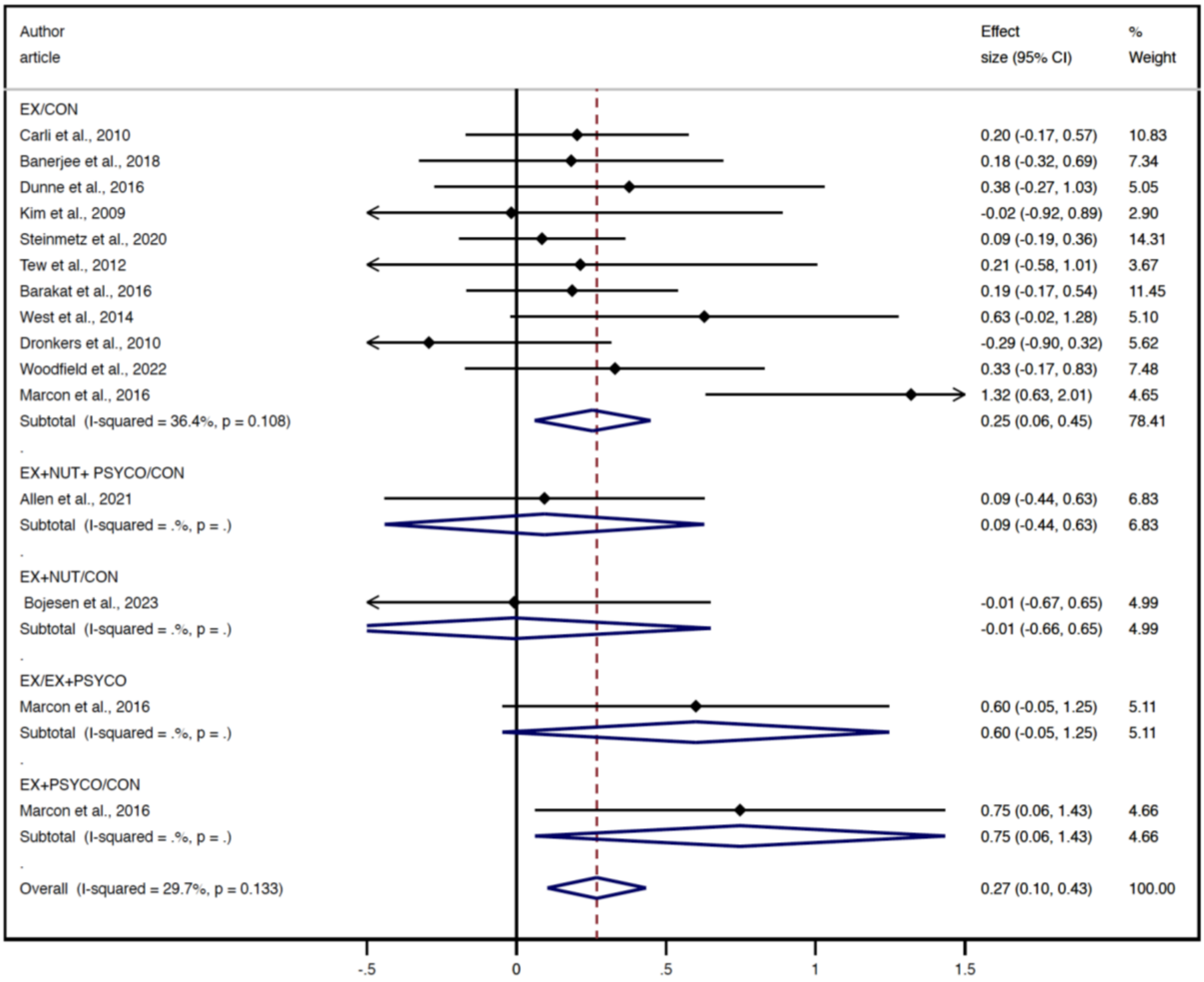


**Figure S.5.** Pooled estimated effect size for VO2 presurgery. Note: Positive effect size (ES) values indicate higher scores in outcomes in favour of the intervention group. **EX:** exercise; **NUT**: nutrition; **PSYCO:** psychological intervention.

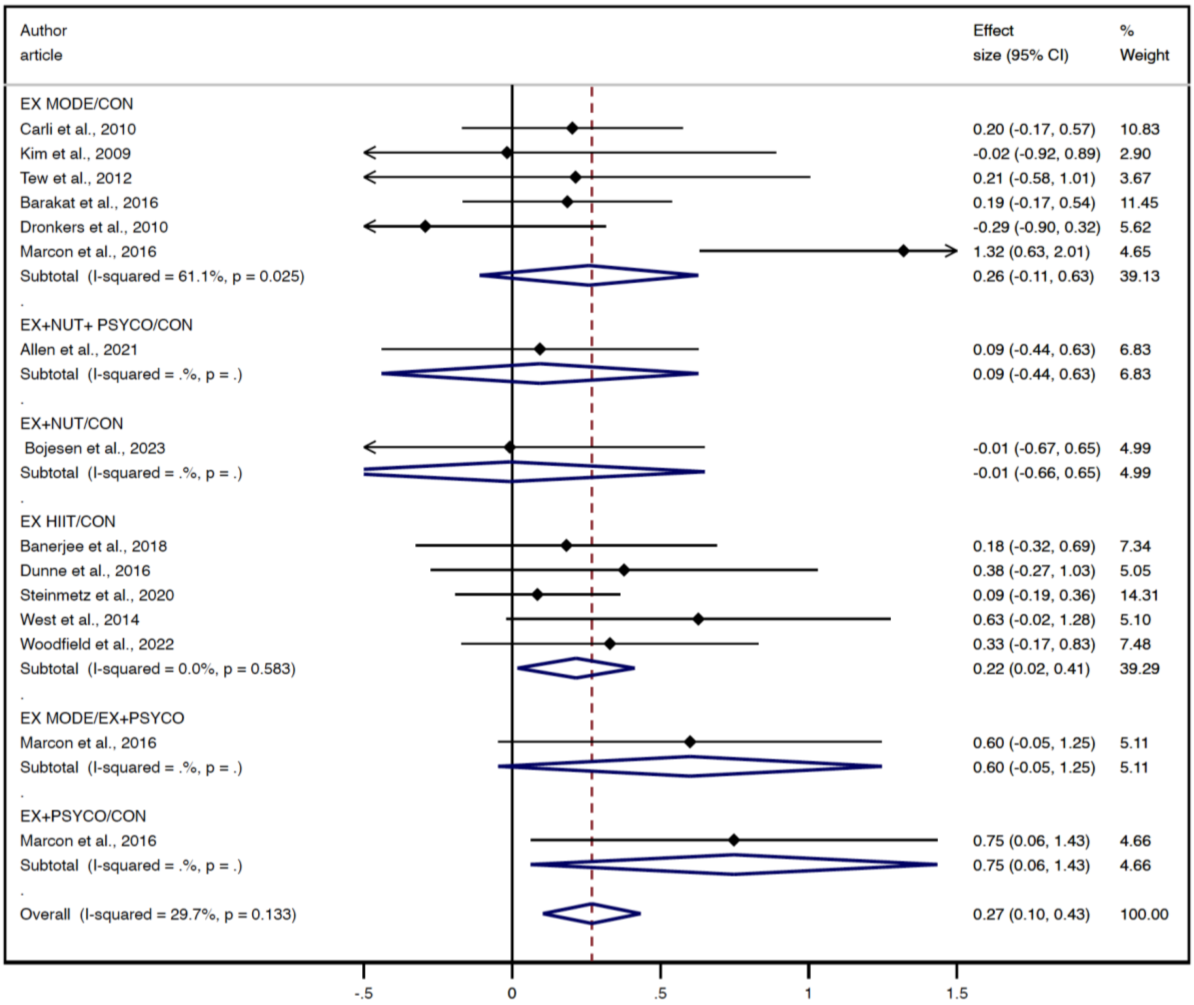


**Figure S.6.** Pooled estimated effect size for VO2 presurgery, subclassifying exercise according to intensity. Note: Positive effect size (ES) values indicate higher scores in outcomes in favour of the intervention group. **EX:** exercise; **HIIT:** high-intensity interval training; **MODE:** moderate-intensity training; **NUT**: nutrition; **PSYCO:** psychological intervention.

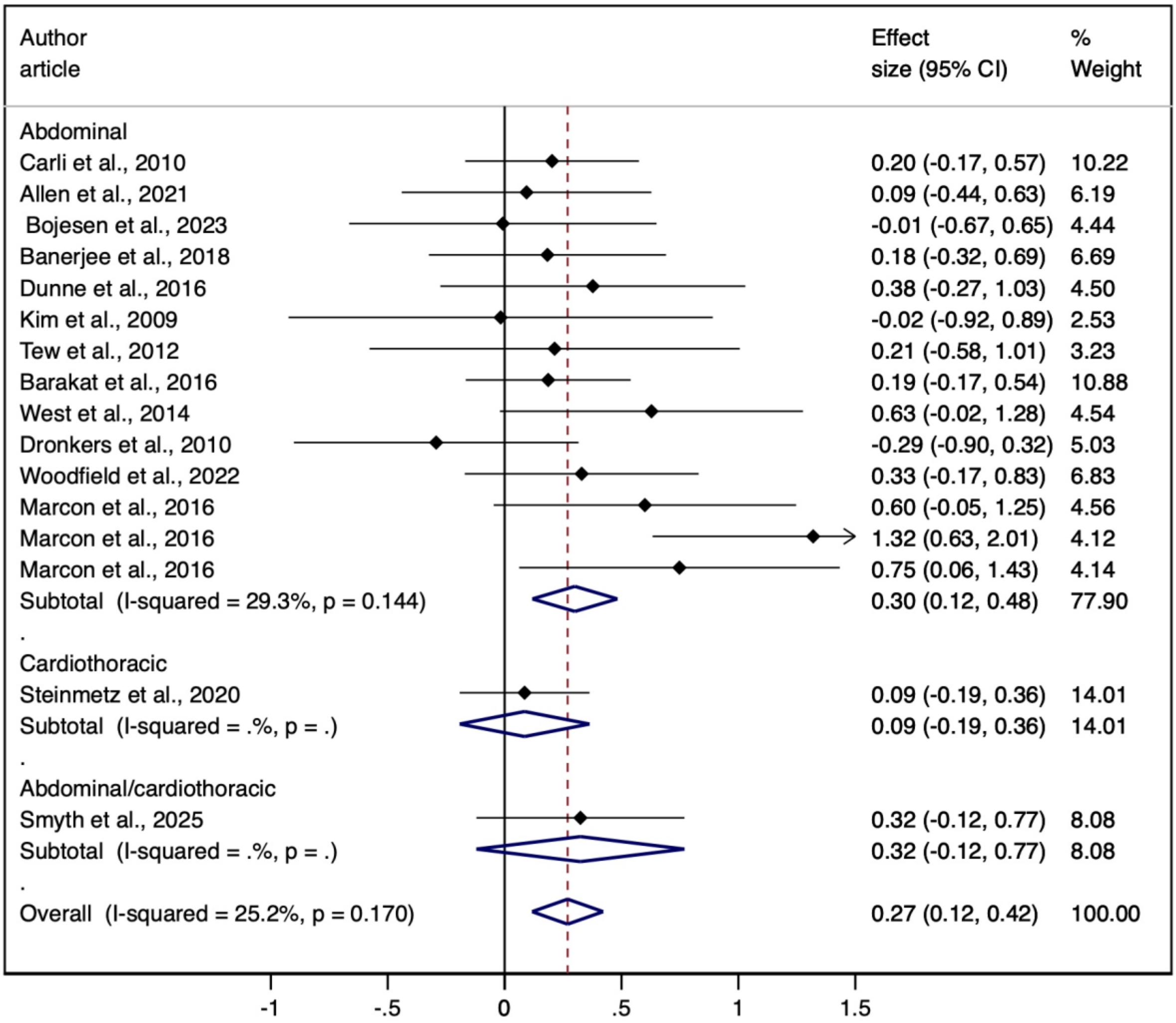


**Figure S.7.** Pooled estimated effect size for VO2 presurgery depending on the type of surgery. Note: Positive effect size (ES) values indicate higher score in outcomes in favor of the intervention group.

**Table S.12.** Subgroups analyses based on the type of surgery.

| **Type of surgery** |
|---|
| The pooled ES for **abdominal** surgery was 0.30 (95% CI 0.12-0.48). For **cardiothoracic** the pooled ES estimate was 0.09 (95% CI -0.19-0.36). Foa abdominal/cardiothoracic the pooled ES estimate was 0.32 (95% CI -0.12-0.77). (Figure S7). |

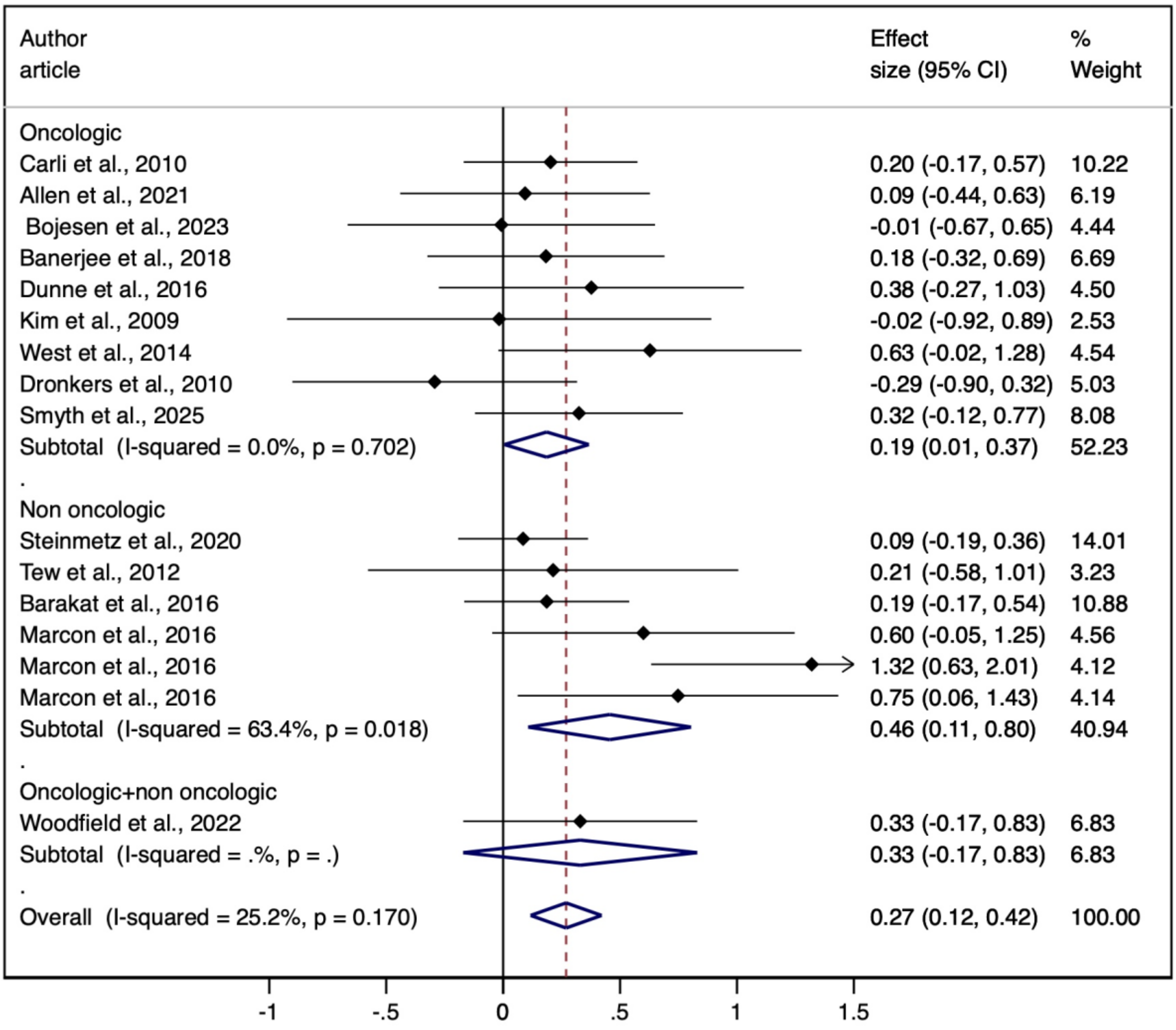


**Figure S.8.** Pooled estimated effect size for VO2 presurgery depending on the type of pathology oncologic or non-oncologic. Note: Positive effect size (ES) values indicate higher score in outcomes in favor of the intervention group.

**Table S.13.** Subgroups analyses based on the type of pathology.

| **Type of pathology** |
|---|
| The pooled ES for **oncologic pathology** was 0.19 (95% CI 0.01-0.37). For **non-oncologic** the pooled ES estimate was 0.46 (95% CI 0.11-0.80). For **oncologic/non-oncologic** the pooled ES estimate was 0.33 (95% CI -0.17-0.83). (Figure S8). |

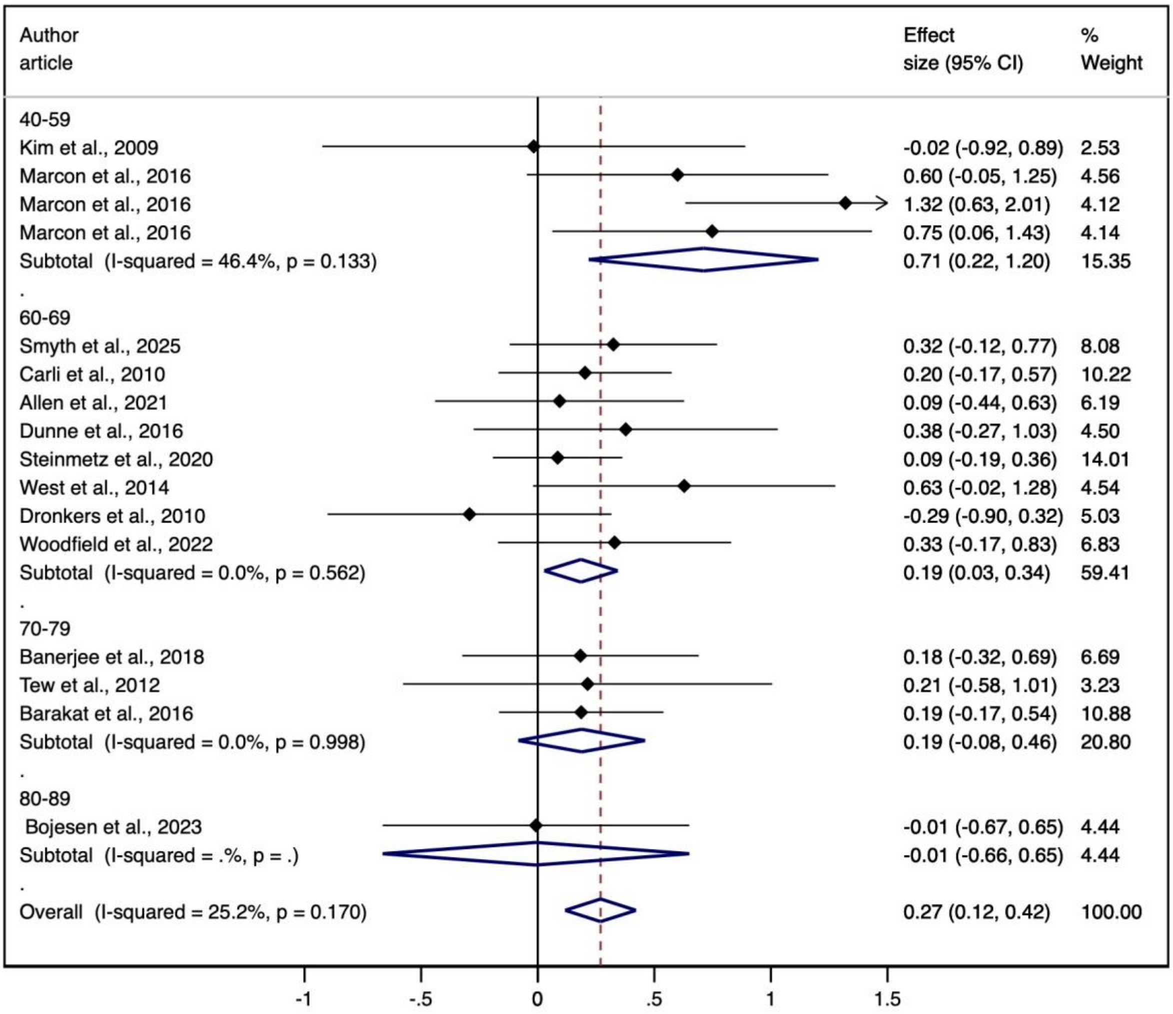


**Figure S.9.** Pooled estimated effect size for VO2 presurgery depending on the age range of population. Note: Positive effect size (ES) values indicate higher score in outcomes in favor of the intervention group.

**Table S.14**. Pooled estimated effect size for VO2 presurgery depending on the age range of population. Note: Positive effect size (ES) values indicate higher score in outcomes in favor of the intervention group.

| **Age range of the population.** |
|---|
| The pooled ES for population **40-59 years** was 0.71 (95% CI 0.22-1.20). For **60-69 years** the pooled ES estimate was 0.19 (95% CI 0.03-0.34), for **70-79 years** the pooled estimate was 0.19 (95% CI -0.08-0.46), and for **more than 80 years (80-89)** it was -0.01 (95% CI -0.66- 0.65) (Figure S.9). |

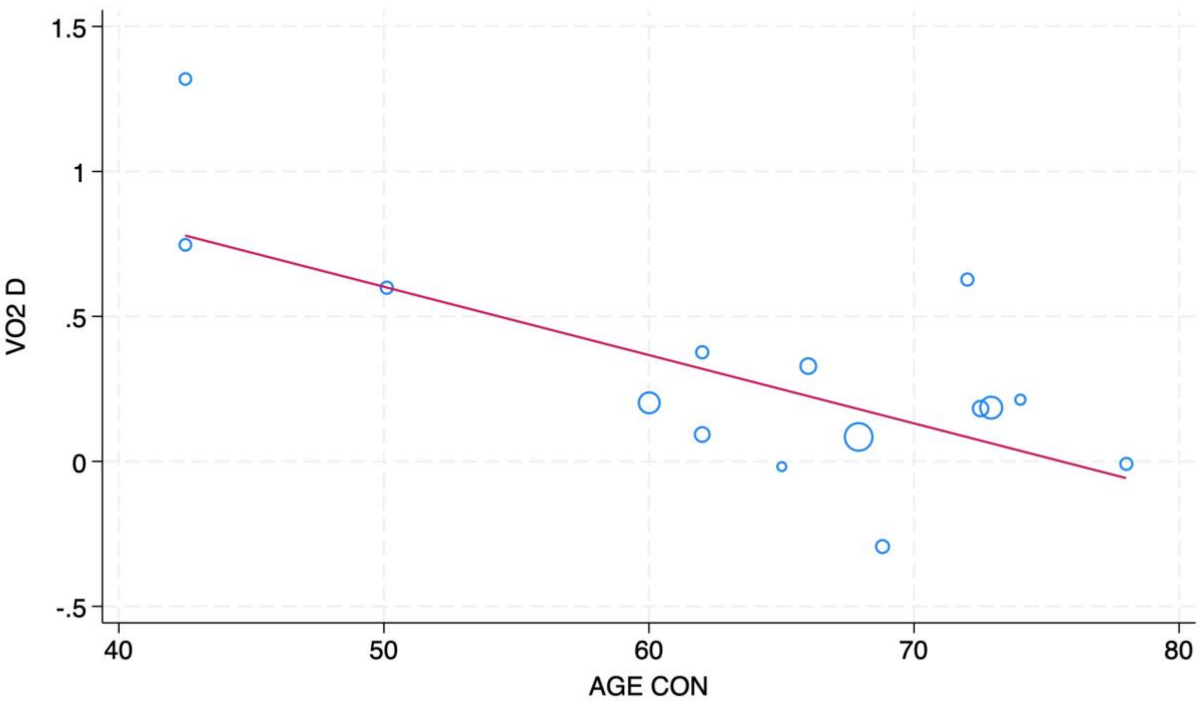


**Figure S.10.** Meta-regression for VO2 presurgery.

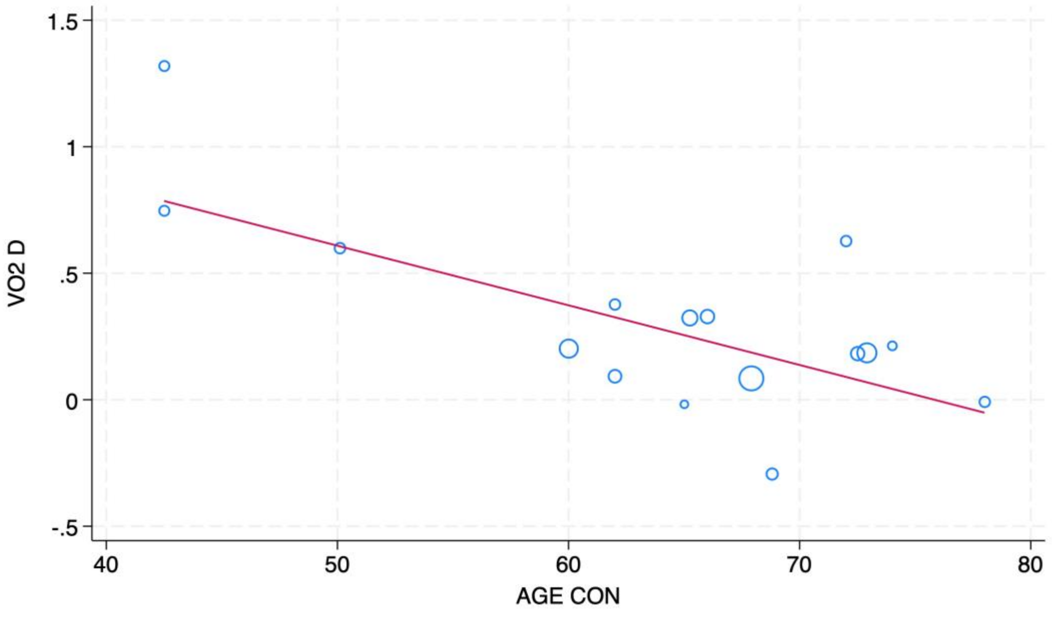


**Figure S.11.** Meta-regression for VO2 presurgery subclassifying exercise according to intensity.

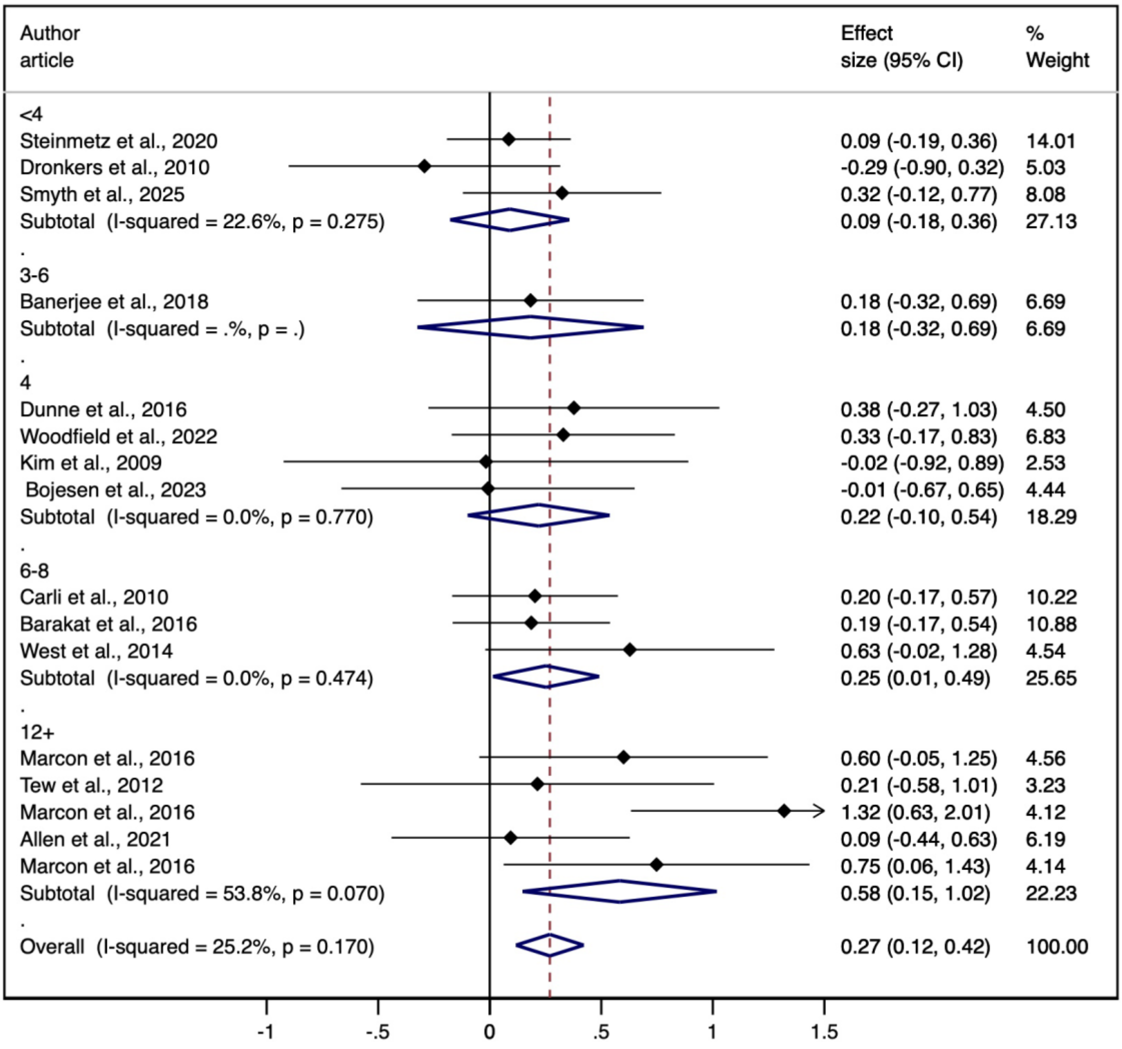


**Figure S.12.** Pooled estimated effect size for VO2 presurgery depending on the duration of the intervention. Note: Positive effect size (ES) values indicate higher score in outcomes in favor of the intervention group.

**Table S.15.** Subgroups analyses based on the duration of intervention.

| **Duration of intervention.** |
|---|
| The pooled ES for <4 **weeks** was 0.09 (95% CI -0.18-0.36). For **3-6 weeks** the pooled ES estimate was 0.18 (95% CI -0.32-0.69), for **4 weeks** the pooled ES estimate was 0.22 (95% CI -0.10-0.54), for **6-8 weeks** it was 0.25 (95% CI 0.01- 0.49) and for more than 12 weeks, 0.58 (95% CI 0.15-1.02) (Figure S12). |

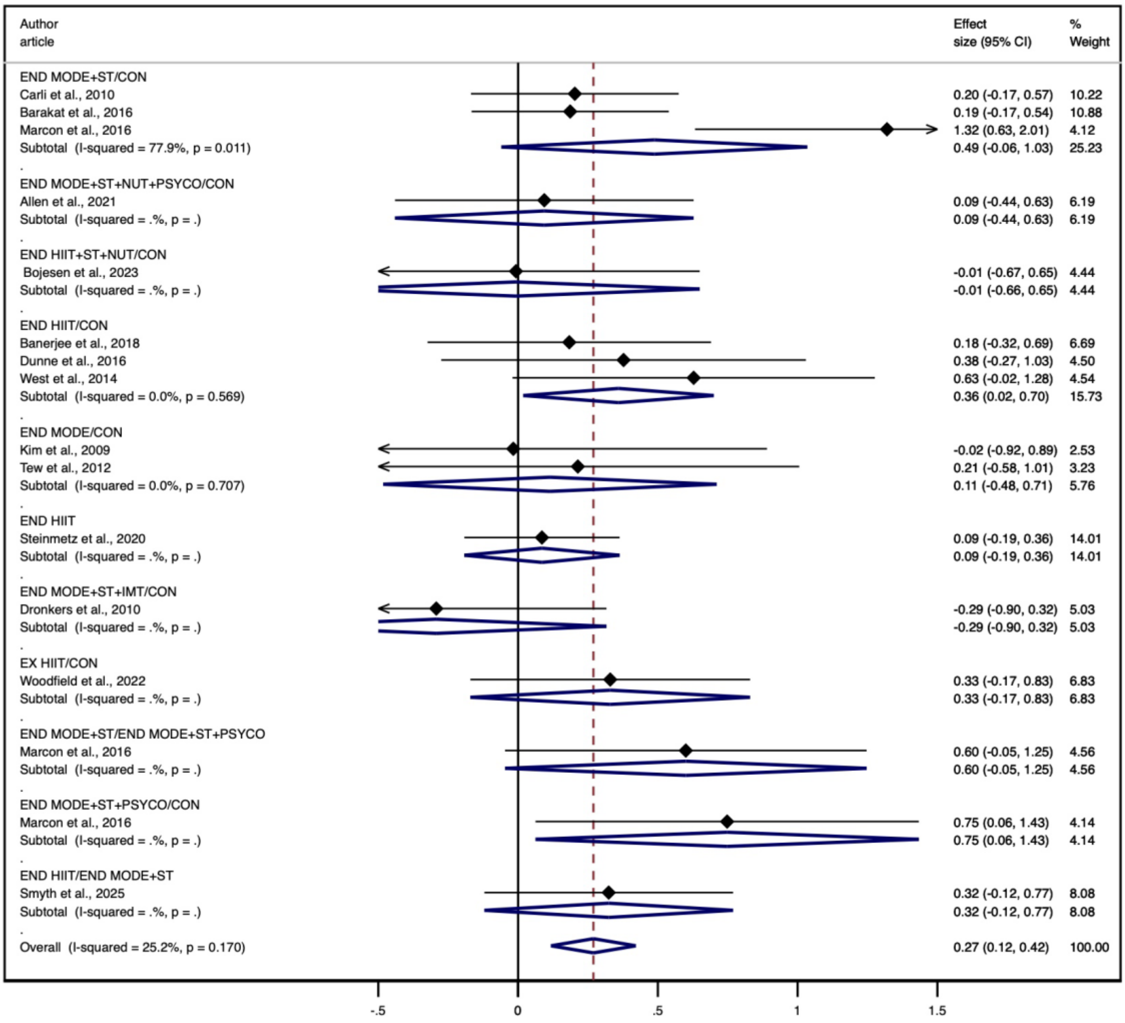


**Figure S.13.** Pooled estimated effect size for VO2 presurgery depending on type of exercise. Note: Positive effect size (ES) values indicate higher score in outcomes in favor of the intervention group. **END:** endurance; **EX:** exercise; **IMT:** inspiratory muscle training; **ST:** strength.

**Table S.16.** Heterogeneity statistics for each pairwise comparison (VO2 presurgery)

| Intervention | Q (df) | p | $I^2$ | $\tau^2$ |
|---|---|---|---|---|
| EX vs CONTROL | 15.73 | 0.108 | 36.4 | 0.0360 |
| EX+NUT+PSYCO vs CONTROL | 0.00 | 0.000 | 0.0 | 0.0000 |
| EX+NUT vs CONTROL | 0.00 | 0.000 | 0.0 | 0.0000 |
| EX vs EX+PSYCO | 0.00 | 0.000 | 0.0 | 0.0000 |
| EX+PSYCO vs CONTROL | 0.00 | 0.000 | 0.0 | 0.0000 |

**EX:** exercise; **NUT**: nutrition; **PSYCO:** psychological intervention.

**Table S.17.** Heterogeneity statistics for each pairwise comparison (VO2 presurgery, subclassifying exercise according to intensity).

| Intervention | Q (df) | p | $I^2$ | $\tau^2$ |
|---|---|---|---|---|
| EX MODE vs CONTROL | 12.87 | 0.025 | 61.1 | 0.1197 |
| EX+NUT+PSYCO vs CONTROL | 0.00 | 0.000 | 0.00 | 0.0000 |
| EX+NUT vs CONTROL | 0.00 | 0.000 | 0.00 | 0.0000 |
| EX HIIT vs CONTROL | 2.85 | 0.583 | 0.00 | 0.0000 |
| EX MODE vs EX+PSYCO | 0.00 | 0.000 | 0.00 | 0.0000 |
| EX+PSYCO vs CONTROL | 0.00 | 0.000 | 0.00 | 0.0000 |
| EX HIIT vs EX MODE | 0.00 | 0.00 | 0.00 | 0.0000 |

**EX:** exercise; **NUT**: nutrition; **PSYCO:** psychological intervention; **HIIT:** high-intensity interval training; **MODE:** moderate-intensity training.

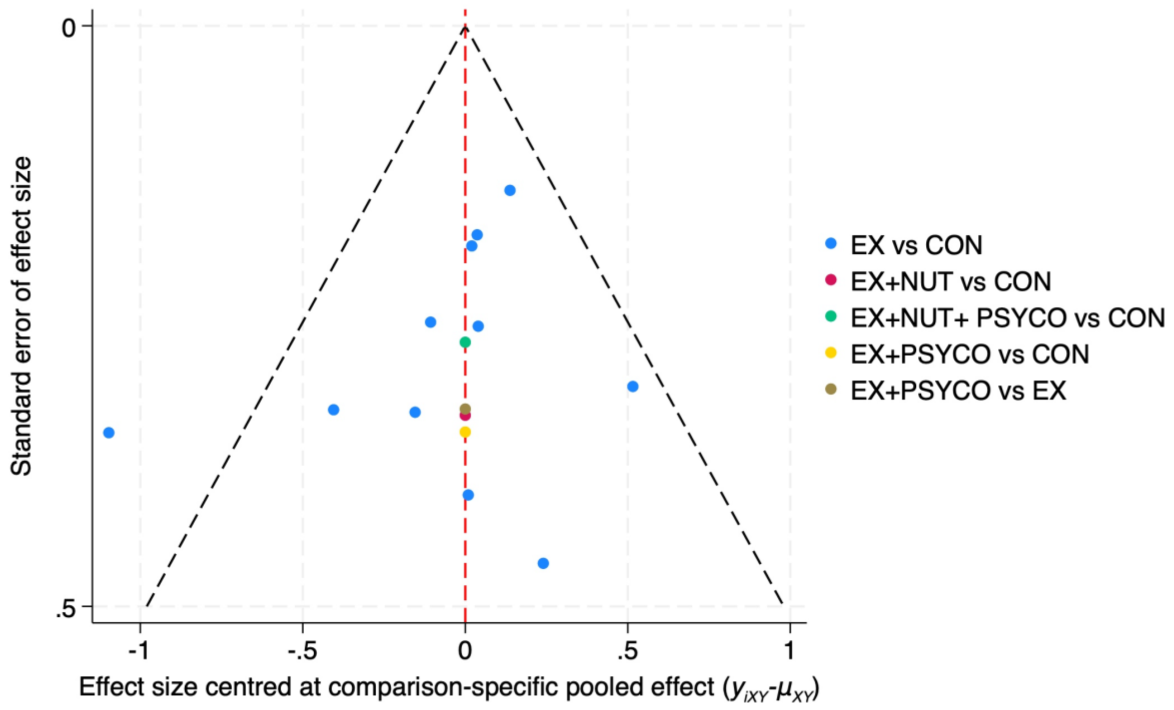


**Figure S.14.** Funnel plot for comparison-specific pooled mean differences in VO2 presurgery **EX:** exercise; **NUT**: nutrition; **PSYCO:** psychological intervention**.**

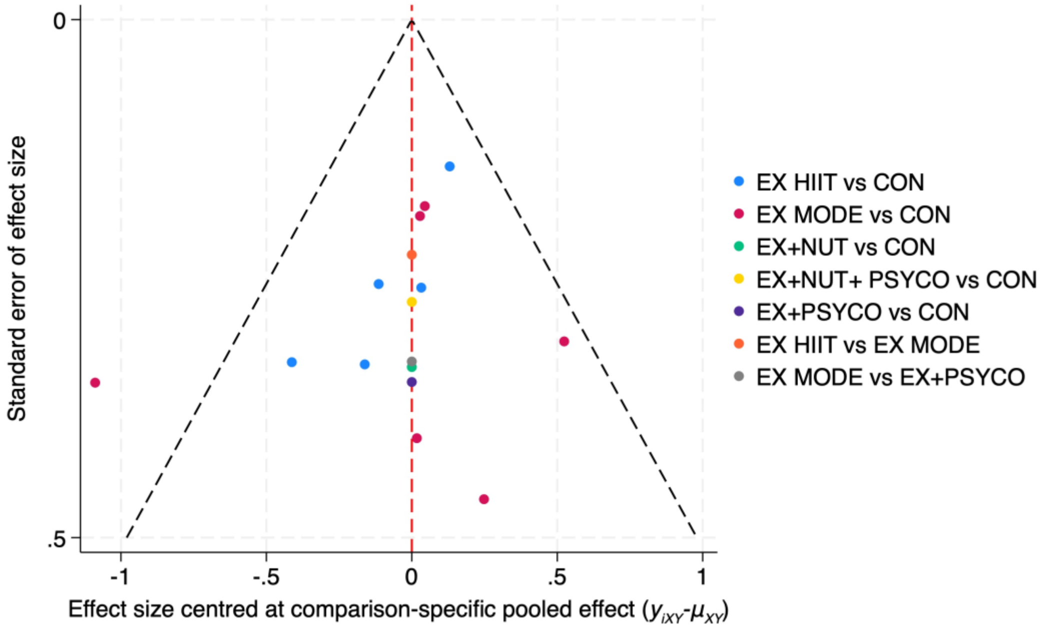


**Figure S.15.** Funnel plot for comparison-specific pooled mean differences in VO2 presurgery, subclassifying exercise according to intensity. **EX:** exercise; **NUT**: nutrition; **PSYCO:** psychological intervention**.**
